# Secure and Time-Aware Communication of Wireless Sensors Monitoring Overhead Transmission Lines

**DOI:** 10.3390/s17071610

**Published:** 2017-07-11

**Authors:** Katarzyna Mazur, Michal Wydra, Bogdan Ksiezopolski

**Affiliations:** 1 Faculty of Mathematics, Physics and Computer Science, Maria Curie-Sklodowska University, Lublin 20-031, Poland; 2 Faculty of Electrical Engineering and Computer Science, Lublin University of Technology, Lublin 20-618, Poland; m.wydra@pollub.pl; 3 Department of Informatics, Polish-Japanese Academy of Information Technology, Warsaw 02-008, Poland; bogdan.ksiezopolski@acm.org

**Keywords:** wireless sensor networks, smart grid, overhead transmission lines, security, reliability

## Abstract

Existing transmission power grids suffer from high maintenance costs and scalability issues along with a lack of effective and secure system monitoring. To address these problems, we propose to use Wireless Sensor Networks (WSNs)as a technology to achieve energy efficient, reliable, and low-cost remote monitoring of transmission grids. With WSNs, smart grid enables both utilities and customers to monitor, predict and manage energy usage effectively and react to possible power grid disturbances in a timely manner. However, the increased application of WSNs also introduces new security challenges, especially related to privacy, connectivity, and security management, repeatedly causing unpredicted expenditures. Monitoring the status of the power system, a large amount of sensors generates massive amount of sensitive data. In order to build an effective Wireless Sensor Networks (WSNs) for a smart grid, we focus on designing a methodology of efficient and secure delivery of the data measured on transmission lines. We perform a set of simulations, in which we examine different routing algorithms, security mechanisms and WSN deployments in order to select the parameters that will not affect the delivery time but fulfill their role and ensure security at the same time. Furthermore, we analyze the optimal placement of direct wireless links, aiming at minimizing time delays, balancing network performance and decreasing deployment costs.

## 1. Introduction

**Figure 1:**
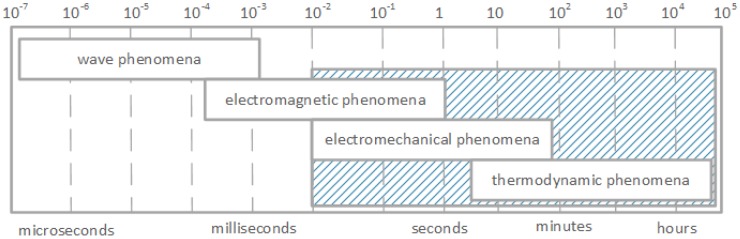
The time frames of the basic power system dynamics phenomena together with corresponding WSN monitoring capabilities.

A smart grid is an initiative to promote and transform the traditional power systems to modern and automated power grids. An electrical power system is a set of many elements such as transmission lines, transformers and generators connected together into a larger system, that can generate, transmit and distribute electric power. Different kinds of electrical elements imply a large variety of dynamic actions or responses to disturbances. Some power system disruptions can affect single element, others can affect larger fragments. Some failures can spread and affect the system as a whole. As each dynamic effect reflects certain unique feature of power system dynamics, some of them can be grouped according to their cause, consequence, time frame, physical character or the place in the system that they occur. Based on the physical character of the disturbance, different power system dynamics can be divided into four groups, defined as: wave, electromagnetic, electromechanical and thermodynamic [1]. This classification also corresponds to the time frame involved (Figure 1 and Table 1). The fastest transients are related to the wave effects or surges in high voltage transmission lines and correspond to the propagation of electromagnetic waves caused by lightning strikes or switching operations. The time frame of these dynamics ranges from microseconds (μs) to microseconds (ms).

Much slower are the electromagnetic dynamics, that take place in the machine windings following a disturbance, such as a short-circuit, operation of the protection devices like the distance or overcurrent protection as well as the interaction between the electrical machines and the power system. Their time frame ranges from ms to seconds (s).

Due to the oscillation of the rotating masses of the generators and motors that occurs usually after disturbance like a short-circuit or switching off large amount of generation, the electromechanical dynamics are even much slower. Electromechanical transients are also caused by operation of the protection system such as underfrequency or undervoltage protection. The time frame of these dynamics is from around a second to several seconds.

The slowest dynamics are the thermodynamic transients, related to the active power generation control in power plants (boiler-turbine-generator control) or transmission line wires temperature variations due to varying weather conditions and line current flow. (The line current flow is a consequence of weather conditions or disturbances like long-term line overloading because of high ambient temperatures, short-term line overloading due to power swings in the system or very short-term high overcurrent due to short-circuit.)

A careful inspection of Figure 1 shows the classification of power system dynamics with respect to the time frame. As it is evident from Figure 1 , they are closely related to where the dynamics occur within the system. For instance, moving from left to right along the time scale in Figure 1 corresponds to moving through the power system from the electrical circuits (that contains elements such as resistance (or resistor), inductance (inductive coil or solenoid) and capacitance (or capacitor)) of the transmission network, through the generator armature windings to the field and damper winding, then along the generator rotor to the turbine until finally the thermodynamics of boiler-turbine-generator slow transients are reached [1].

In today’s electric power systems, sensing and monitoring equipment is employed in a limited number of critical assets, such as power transmission lines and substations. Generally, this sensing equipment is not interconnected, and transmits the collected data to a central location using wire communications (such as Ethernet or fiber-optic), where a supervisory control system and dispatchers at the utility headquarters manage grid operations. Wired monitoring systems require expensive communication cables to be installed and regularly maintained. Additionally, the use of wire communication in harsh environments is very often uneconomical or even impossible. Hence, there is an urgent need for cost-effective wireless monitoring and diagnostic systems that can improve system reliability and efficiency by optimizing the management of electric power systems. Recent studies showed [2,3], that the philosophy of WSNs has a great potential to address the challenges of the existing power grids and fulfill their sensing and communication tasks. The need for uninterrupted electricity supply and the high costs of building new generating and transmitting power facilities are forcing governments and operators to make an efficient use of network infrastructure and increase the regulatory abilities of power grids using new kinds of measurements. Certain and uninterrupted supply of electrical energy became the foundation for the creation of a new Information and Communication Technologies (ICTs) [4] (such as WSN measurement systems). The ability to harvest new information about transmission line parameters implied a rapid development of new algorithms for power systems operation. One way of more efficient usage of grid infrastructure (for instance, overhead transmission lines and increasing its ampacity and transmission flexibility) is the monitoring of weather conditions, mechanical and electrical parameters along lines in order to determine the Dynamic Line Rating (DLR) and allow for controlled overloading during certain time. Additional measurements of the plurality of non-electrical quantities such as temperature, wire strain elongation or sag with the current weather conditions pose an additional information about safe or unsafe operation of transmission lines. Above considerations lead to the conclusion that WSN can provide an important information, which can be used for power system estimation or optimization along with providing an additional decision variable for dispatcher to turn off the line or not.

Since monitoring and control of electric power systems are essential for their efficient and effective functioning, WSNs, as a low-cost, flexible and self-organizing devices, appear to be the perfect choice for creating a highly reliable and self-healing smart power grid that rapidly responds to online events with appropriate actions. The nature of WSNs brings significant advantages over traditional communication technologies. WSNs are characterized by a rapid and straightforward deployment (even in difficult-to-access and large geographical locations), high coverage area, low installation and maintenance costs, as well as easy replacement and upgrading procedures. Furthermore, they have the capability to organize and configure themselves into effective communication networks. Their flexibility and mobility is higher than the one of the wired networks. Potential applications of WSNs on smart grid span a wide range, including, but not limited to: power quality monitoring, outage detection, overhead transmission line monitoring (such as conductor elongation or temperature measurements in dynamic load rating systems), fault detection and location, equipment fault diagnostics and underground cable system monitoring [5]. The collaborative and context-aware nature of WSN brings several advantages over traditional sensing including greater fault tolerance, improved accuracy, larger coverage area, and extraction of localized features. Despite undeniable advantages, there are also several challenges, such as security. As is the case with all kinds of wireless networks, WSNs are more vulnerable to security threats originating from the open communication environment, which pose serious risks and expose the system to many threats. Unlike other wireless technologies, applying advanced and complex security mechanisms to WSN is not applicable due to their physical limitations. This complicates protection measures and makes WSNs more vulnerable to external attacks. Moreover, WSN-based smart grids suffer from all the security threats facing classical WSN communication. Because of their complexity, large number of stakeholders, and highly time-sensitive operational requirements, power grids have additional vulnerabilities and are prone to varied types of attacks [6,7,8]. One should also remember, that available security solutions, even those designed for WSNs, are also very often costly and consume large amounts of the Central Processing Unit (CPU). Another drawback of using WSNs on long distances, are time delays. The delays in information transmission depend, among others, on the possibility of practical use of the structure of WSN to/for the DLR or to support the work of securing or blocking its action. Remaining delays are related to utilized routing algorithms and depend on how far the data needs to travel through the transmission grid and the time when the collected data is finally sent to the base station. For environments with rigorously defined requirements on data delivery, it is essential to minimize the information transmission delay in order to allow for efficient and effective communication. Hence, in this paper, we focus on minimizing delay time considering the reliability of the communication simultaneously. To overcome the above issues, we proposed the secure and reliable communication plan for WSN in transmission grid systems.

In this paper, we contribute to analyzing and solving the problem of time delays generated during measurement data transmission. The main contributions of this paper are summarized as follows.

- In order to find a trade-off between the security, performance and time delays, we design a reconfigurable network model and demonstrate a feasible analysis considering various practical issues, concerning routing operation of the WSN and the security of the data traversing transmission grid.
- We present a case study, in which we develop a smart grid model in Quality of Protection Modeling Language (QoP-ML), in order to better understand the relation between transmission delays, routing algorithms and different transmission grid architectures and test our ideas in practice. Through a set of simulations, we try to identify the optimal positions of direct connections, in order to minimize transmission delay, energy consumption and financial cost of sensors’ arrangement.
- We introduce a secure and time-aware protocol to ensure communication security. Additionally, we try to answer questions about the confidentiality, integrity, and availability of the data traversing smart grid environments.

The remainder of this paper is organized as follows: in Section 2, we present the related work. Moving on to Section 3, we discuss our environment, describing considered architectures and scenarios. Section 4 is focused on practical representation of our approach. Next, in Section 5, we analyze the results and investigate the relationship between time delays and different architectures. Finally, we conclude with Section 6, in which we summarize our work.

## 2. Related Work

The smart grid can provide efficient, reliable, and safe energy automation service with two-way communication and electricity flows. The use of WSN helps in capturing and analyzing data related to power usage, delivery, and generation. The energy usage and management information, including the energy usage frequency, phase angle and the values of voltage, can be read in real time from remote devices. Therefore, utility companies can manage electricity demand efficiently. They can reduce operational costs by eliminating the need for human readers and provide an automatic pricing system for customers, who can enjoy highly reliable, flexible, readily accessible and cost-effective energy services. However, in order to achieve a trade-off between the performance and potential time delays, the analyzed architecture should be carefully examined.

Given the vast geographical expanse of the transmission grid architecture, delivering monitoring information to the control center in a cost efficient and timely manner is a critical challenge to be addressed in order to build an intelligent smart grid. Since WSNs present a feasible and effective solution for transmission of monitoring data, there exist many works dealing with the possibility of adopting WSN for the purposes of monitoring the operational parameters of power lines [9,10,11,12]. Although optimization of WSN architectures monitoring transmission lines has been examined many times before [2,3,13,14], there was no exact analysis concerning security of provided solutions.

In [13] the authors propose a hybrid (combined of wired (copper cable/optical fiber and wireless (cellular/IEEE 802.15.4) standards) hierarchical network to provide cost optimized delay and bandwidth constrained data transmission. They formulate a placement problem in order to optimize the number and location of the cellular enabled towers (base stations) to significantly reduce the operational and installation costs while respecting effective data transmission, bandwidth, delay and connectivity constraints. Their goal was to minimize the installation and operational cost while satisfying the latency and bandwidth constraints of the data flows. Researchers evaluate proposed solution (using the ILOG CPLEX 12.2 software for the simulation), considering an example transmission line network with defined number of towers, average span, data packet length and bandwidth. They examine different scenarios, including variation in flow bandwidth, network size and compare obtained results. Although scientists claim that their method is generic and encompasses variation in several factors, they consider neither the security of the transmission, nor the routing algorithms responsible for traversing the data to the base station.

Another paper, which deals with a problem of efficient communication of sensors monitoring transmission power lines, is presented in [3]. Here, the researchers propose a network model, in which wireless sensors, deployed all over transmission line, communicate with each other in order to (if needed) reconfigure (based on the application requirements) to deliver information to substations efficiently and effectively. The authors examine the performance of the linear network model, providing calculations using sensors with defined characteristics. Then, they present an example reconfigurable network model and illustrate its usage with a simple case study. In the article, scientists emphasize on transmission time delays, mentioning neither energy consumption, nor security.

[2] consists of extended research proposed in [3]. In this paper, authors study how the data measured on transmission power lines can be delivered efficiently to substations. They try to find the optimal placement of direct wireless links, aiming at minimizing the delay in information delivery. First, researchers again consider the linear network model and prove that it cannot deliver information in a timely fashion and suffers from an imbalance of workload, because the relays closer to the substations have to handle a lot more traffic that those which are located farther away. Therefore, they formulate the general solution for minimizing the delay of data delivery. The main idea is to divide all the nodes into different groups, where each group contains the nodes that send information to the same base station. Finally, using introduced formulas for optimal placement of direct links, researchers provide numerical results and analyze how the number of groups influences time delay and energy consumption. Improvement in minimizing time delays achieved by the proposed method is significant. However, focusing on optimal transmission time, scientists do not consider the security of the transmission.

As recent studies show [2,3,13], the idea of using WSNs in power grids is an important issue. However, none of the works examined above, pays attention to the security of the data traversing through wireless network. Since radio waves in a wireless communication spread in the air, one common risk is that wireless channels are more insecure and susceptible to numerous attacks than wired networks. This is why the security of the electrical transmission power grid using wireless sensor networks is one of the most essential factors in this type of deployments. In this paper, we try to face this problem, considering not only the minimization of time delays, energy consumption and the financial cost, but due to the data sensitivity, also take into account the security of the transmission.

## 3. Transmission Line Monitoring Using WSN in Smart Grids

In this section, we describe a transmission line monitoring scheme, proposing an example architecture of a WSN deployed in miscellaneous places of considered transmission line. Moreover, we develop corresponding scenarios, in which we examine the performance, security, the transmission time and delays in defined conditions, providing concise representation of utilized routing algorithms and packet flows. Through a set of simulations, we analyze gathered results to identify the optimal routing scheme in the existing WSN, in order to minimize the transmission time delay. Using gathered results, we try to answer the question, which type of a WSN architecture, along with routing algorithms and security mechanisms, can be used for measuring and monitoring power system phenomena, and is capable of acting as an additional measure of protection, control or operational optimization.

### 3.1. Smart Grid Architecture

**Figure 2:**
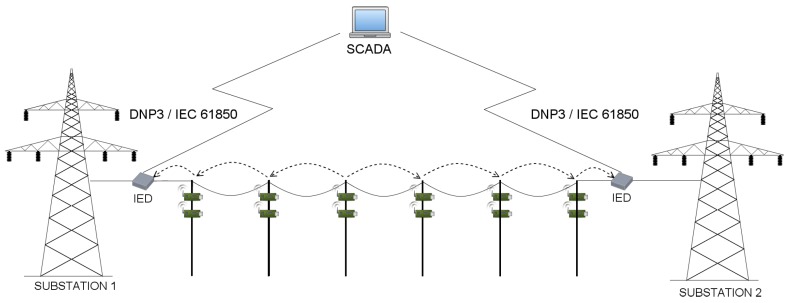
A typical transmission grid environment.

In general, power grid energy subsystem is divided into power generation, transmission, distribution and consumer site. On the other hand, the power grid communication system is composed of several subsystems, being in fact a network of networks. The core of the monitoring and control of a substation is the Supervisory Control And Data Acquisition (SCADA) system. Remaining communication networks in smart grid systems include, for instance, cellular, microwaves, fiber optics, serial links or wireless local area networks. As in this paper we try to answer questions about time delays generated during measurement data transmission, we consider only the transmission grid. A typical transmission line is presented in Figure 2. Within the transmission grid, WSNs deal with power transmission issues and distribution monitoring. Because sensors handle sensitive and confidential power grid data, which should be securely transmitted to prevent from the theft and to avoid any unauthorized access, secure and reliable protocols need to be implemented. Because the nature of WSN makes the system vulnerable to various attacks, physical and cyber threats, we decide to analyze various WSN deployments (which differ in utilized security mechanisms or routing algorithms) and indicate the architecture, that will be the most suitable for different smart grid applications.

**Figure 3:**
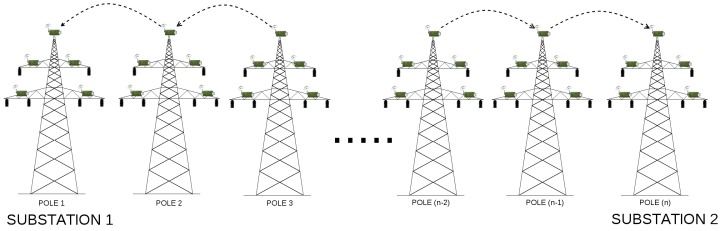
Default, hop-by-hop WSN architecture deployed on the example transmission grid.

**Figure 4:**
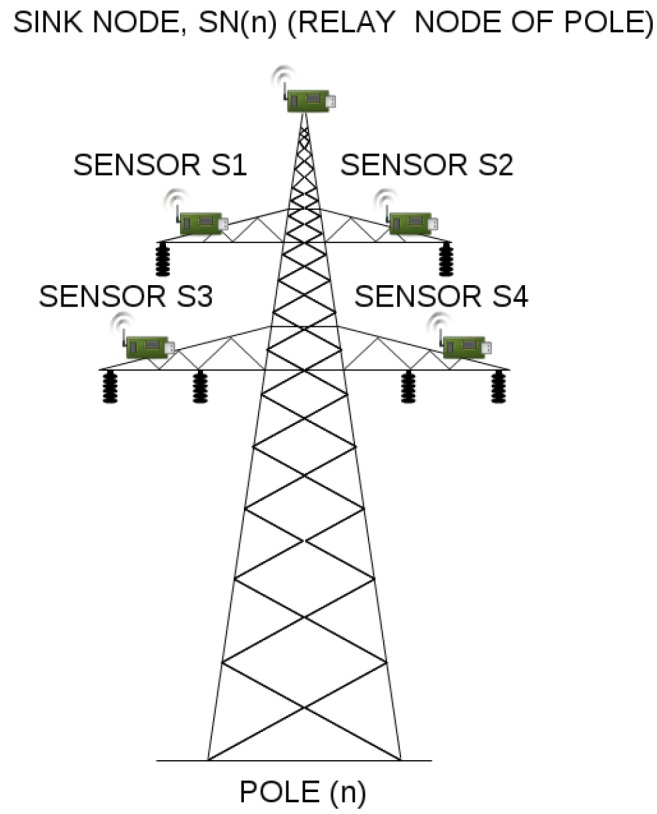
An electric pole with wireless sensors deployed.

An example, 110 kV transmission line, with wireless sensors deployed in miscellaneous places of electric poles (which is the basis of our considerations) is presented in Figure 3. Poles connect transmission lines between two substations. When considering 110 kV transmission lines, the most common distance between two poles ranges between 100 and 300 m. There exist however, transmission lines with the distance between poles equal to 450 m, but these are 400 kV lines, not considered in our case study. In our scenarios, we assumed the distance between two adjacent poles to be equal to 50, 100, 200 and 300 m. There are 43 poles between two substations, and 45 poles in total (including substations). There are 4 sensors and the relay node (also referred to as the sink node) deployed on each pole along the line (Figure 4), which results in 180 deployed sensor devices in total. A group of 4 sensors send the information to the relay on the same pole. After collecting information from all its slave sensors, the sink node on each pole sends the information to the substation. The role of the substation is to transmit the data to the gateway.

Because the distance between sensors and their relay node is quite small, so a short-range communication technology suffices, we utilized TelosB [15] devices as those located on electric poles and collecting the data (their outdoor range is equal to about 100 m), and Mica2 [16] motes as sink nodes, since their outdoor range is equal to about 300 m (which is quite enough when we consider the distance between two adjacent poles to be equal to 50, 100, 200 and 300 m). Table 2 shows some of the hardware characteristics of the utilized sensor devices.

Mica 2 motes, requiring just 58 mm × 32 mm of silicon, include data RAM, processing, and communication capabilities. The node combined with a tiny battery is capable of periodically reporting its presence for years to come being a a notable example of a general-sensing-class device. It includes a large interface connector allowing its attachment to an array of sensors. By providing a large number of I/O pins and expansion options, the Mica2 is a perfect sensor node option for any application where size and cost are not absolutely critical. It is, for example, easily connected to motion detectors and door-and-window sensors as the foundation of a building security system. Moreover, the Mica2 is capable of receiving messages from nodes attached to high-value assets, including personal computers and laptops, at risk of being stolen. Crossbow’s TelosB mote bundles all the essentials into a single platform including: USB programming, capability, an IEEE 802.15.4 radio with integrated antenna, a low-power MCU with extended memory and an optional sensor suite. It offers many features, including, among others, IEEE 802.15.4/ZigBee compliant RF transceiver, 2.4 to 2.4835 GHz, a globally compatible ISM band and 250 kbps data rate.

When it comes to the measured parameters, in the article we assumed that sensors measure the following quantities: wind speed and direction, atmospheric temperature, pressure and humidity, solar radiation along with conductor current, temperature, tension and inclination. For each pole along the transmission line, each sensor performs 6 measurements (ambient temperature, atmospheric pressure and humidity, solar radiation, wind speed and wind direction are taken into account). Because of the time stamp attached to each measured value, the number of measurements on a pole is doubled, which results in 12 measurement values. There are also 3 current measurements for every phase along with 3 conductor temperature measurements, 4 tension and 4 inclination measurements (for every phase and lighting wire), doubled because of the time stamp. This results in 40 measurement values in total. When we assume that a single measurement can be stored as a single float value (8 bytes), then the size of the packet being transmitted between relay nodes is equal to 320 bytes.

The actual cost of the hardware depends on sensor’s capabilities. An approximate cost of TelosB device, utilized in our scenarios, is equal to $182, while the relay node can be bought for about $152. According to the analysis given in [2] and the information provided in Table 2, the cost of the deployment of a WSN is indeed lower than the cost of, for instance, cellular links.

The values measured by sensors are important for many different smart grid applications. Described parameters are used by wire thermal models of DLR systems for calculating actual value of allowable current or power transfer through the line. Such measurements can be also used by the power system estimation or optimization software. The thermal time constant of the power line conductor ranges from 5 to 15 min and depends on the actual weather parameters. Acquiring data from the whole power line spans in real time determines line operational safety. Let us consider, for instance, the conductor temperature measurement. It can be used for blocking an automatic *reclosing* action after clearing a power line short circuit. Usually, the first reclosing time ranges from 0.5 to 1.4 s, while the second reclosing time is much longer and ranges from 10 to 20 s. Described reclosing time can be extended if the conductor temperature exceeds maximum allowable value after short circuit. In this particular case, in order to provide the required protection of the relay in the substation, it is essential to know if the measurement data containing conductor’s temperature can be delivered within the assumed time period.

In order to fulfill their role accurately, transmission line monitoring systems need to have continuously updated information on measured parameters. As a consequence, proper synchronization between wireless sensors is required, so that the quantities measured in different points of the transmission line could be directly comparable. Implemented WSN monitoring system must be able to acquire information on different nodes of a network in a synchronized manner and distribute the data. Given the complexity of the system to which is applied, the problem of measurements synchronization is an enormous challenge in and of itself. The synchronization accuracy requirements can significantly change depending on the constraints of the applications which vary from system to system. In environmental monitoring applications there exist several possible synchronization solutions. One of the available approaches is to use time stamps, which can be further compared and analyzed. However, in practice, with Network Time Protocol (NTP)-based solutions it is sometimes difficult to ensure such mechanisms are in use and correctly configured on all sensors. Another possible approach is to use the so-called trigger signal for synchronous triggering of each measurement. With such an external synchronization, the system under test triggers a synchronization signal. This method allows to assess the measurement system response with a great accuracy. In the proposed scenarios, we simply assumed that the measurements are triggered by the synchronization signal, such that they are made at the same time, and further transmitted to the substation with the help of the chosen routing algorithm.

Designing our scenarios, we assumed that a power line is located in an open area, where relay nodes antennas are rather visible for each other. However, since wireless radio channel puts fundamental limitations to the performance of wireless communications systems (radio channels are extremely random, and are not easily analyzed), modeling the radio channel is typically done in statistical fashion, using mathematical transmission models. Because the path loss is the largest and most varied quantity in the received signal strength (link budget), we decided to use this model in our simulations, in order to calculate the quality of connections between sink nodes (in our model referred to as the *q* value). Among many other factors, the path loss model depends on frequency, antenna height, receive terminal location, relative to obstacles and reflectors and link distance. The *free space path loss* model is a type of path loss model, which represents realistic empirical propagation loss model, and takes into consideration distance and frequency—for this reason, it is the perfect choice for our estimations. In order to calculate exact *q* values utilized in our simulations, we used technical specifications of Mica2 and TelosB sensor devices in order to obtain their physical characteristics (RF power, frequency, receive sensitivity). The exact *q* value calculation formula is given by the following equation:

(1) $$q=(t_{p}-20\times \log_{10}\left(d\right)+20\times \log_{10}\left(f\right)+32.44)\times \left(-1\right)$$

where:

- $t_{p}$ is the RF power of transmitter, given in dBm
- *d* stands for the distance between two adjacent sink nodes, expressed in km
- *f* represents the frequency of the device, in MHz
- 32.44 is the constant value (if *d* and *f* are given in kilometers and megahertz, respectively)

The *q* value defines the quality of the connection and is calculated using the above formula. It is represented as non-negative real number (q={q∈R|q≥0}), where the lower the *q* value, the better the connection quality.

The success of WSN-based monitoring applications requires the delivery of high-priority events to relay nodes without any loss from the original sources to the final destination. For this reason, the issue of a packet loss is another crucial aspect that must be considered while deploying WSNs as a monitoring solution in a transmission power grid. The packet loss can be caused by a number of factors, including signal degradation over the network medium, congestions due to heavy traffic, collisions at link layer or buffer overflows. The transmission distance between sensor nodes and the quality of the connection affect the packet loss as well. There exists a strong correlation between packet loss and signal strength—over long distances, the signal strength fading effect leads to low signal noise ratio. Environmental interferences also contribute to packet loss. The above constraints emphasize the need for improving transmission reliability in WSNs. One of the most common approaches of dealing with the problem of packet loss is the packet retransmission. Packet retransmission requires some additional mechanisms to be implemented in order to trigger the retransmission of a lost packet. For instance, with the acknowledgment (ACK) mechanism, every successfully received packet should be directly acknowledged by the receiver with a notification message. If a sender is not able to recognize the expected notification message, then the packet should be retransmitted. The ACK mechanism offers highest reliability guarantee. It can be used by protocols providing packet reliability on hop-to-hop as well as on end-to-end levels.

Generally, in order to eliminate or minimize the packet loss in the considered transmission grid environment, it is possible to introduce some retransmission mechanisms, or simply increase the signal strength by powering sensors with energy harvesting devices. However, in our case study, we perform only a single round of measurements (being triggered by the synchronization signal), where the measured data is being transmitted on quite short distances. For this reason, we assumed that in our case, the packet loss is almost equal to 0.

### 3.2. Scenarios

After a brief introduction of the considered environment, let us now move on to the discussion about miscellaneous WSN deployments and routing algorithms, which we prepared for our simulations and developed using QoP-ML. We proposed different scenarios, and analyzed them in terms of transmission time delay and security.

As proved in [2], the linear network model proposed for transmission line monitoring in Figure 3 can not deliver information in a timely fashion. For this reason, instead of focusing only on a linear network model, we decided to analyze some other methods of data delivery as well. Instead of using hop-by-hop communication all over the line (Scenario 5), we proposed to divide all the nodes into smaller groups (called clusters) and route information to cluster heads, such that the cluster heads will communicate directly with the substation (Scenarios 1, 2, 3 and 4). A node that does not connect directly to the cluster head, should send its information in a hop-by-hop manner to the cluster head within its group. The selection of the cluster head is determined by its physical position on an electric pole. The node, which is located on the most appropriate position on a pole (in terms of, for instance, the lack of environmental interferences) is always chosen as a cluster head. The routing algorithm has the information about connections between sensor devices and the quality of paths used by devices for sending packets such that they can reach the substation. It also knows about which of them is the cluster head. We denoted the number of all nodes by φ, while the number of hops within a single group (cluster) is denoted as η. Then, the number of groups on power line (or, the number of direct links to base station) is given as:

(2) $$\kappa =\frac{\varphi }{\left(\eta \times 2\right)}\;\mathrm{where}\;\eta \;>\;0\;,$$

and, in our case, η= [1,⋯,23]∩{45}. Since the number of electric poles within the power line is fixed, we can assume that φ is constant and equal to 45. Thus, using the Formula (2), it is fairly easy to calculate κ (see Table 3). As mentioned before, κ values represent the number of wireless, cellular links, which the relay nodes (designated cluster heads) use to send collected data directly to the base station. Below we describe each of the prepared scenarios in greater detail.

***Scenario 1*** Developing the first scenario, we examined the linear network model, assuming that there are 45 poles in total, and the power line has only 2 direct links to the substation. In this type of network (Figure 3), where the data traverses in a hop by hop manner, a relay node not directly connected to the substation sends its information to its neighbour relay, that is closer to the substation. For instance, the relay node on 42nd pole, would send its data to 43rd pole, which can then send its own data, together with the data from pole 42, to pole 44. After reaching the 44th pole, the relay node on this pole sends the data to the substation, which transmits it to the gateway. In order to improve data delivery, we decided to divide considered power line in two sub-lines, where relay nodes on poles 0–21 send the information to the substation on the left side of the power line, while sink nodes on poles from 22 to 44 transmits the data to the second base station, located at the right end of the power line. Due to the fact that the distance between the sensor and the relay node is quite small, so a short-range communication technology suffices, we simply omitted this value in our simulations.

**Figure 5:**
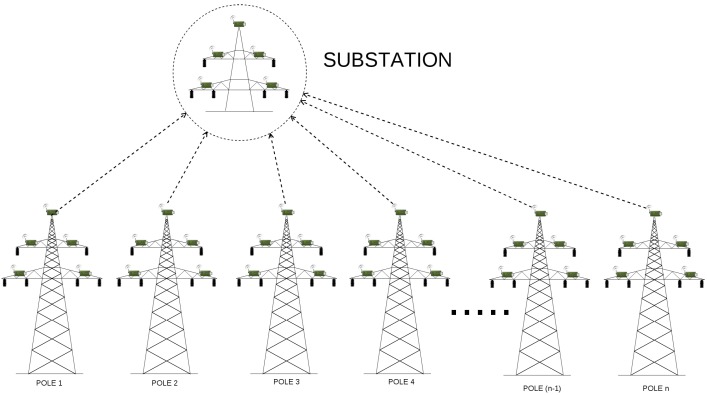
WSN architecture deployed on the example transmission grid, where η=1.

***Scenario 2*** In this scenario, we consider a situation where every relay node on each of 45 poles has direct connection to the substation. Because there are no intermediary relay nodes between the relay node on a pole and the substation, logically this type of deployment should have the smallest delay time of all (Figure 5). By setting up a direct link to the base station on each pole, the delay will be minimized and the workload among relays will be balanced. Nevertheless, such solution requires the base station to be within sensor’s radio range (so they both can hear each other). Although pretty small time delays speak in a favor of this scenario, there exists just another disadvantage that prevents from using it in practice. This approach is expensive in terms of equipment cost and extra energy consumption of the direct cellular wireless links.

***Scenarios 3 and 4*** In order to find a compromise between the cost and time delays, we decided to select only some relay nodes to establish direct links to the base station. Relays that are not directly connected to the base station should send their information to cluster heads which have a direct link to base station established. It is thus obvious, that the positions of direct links would influence time delays. To find the optimal arrangement of direct base station connections, we proposed to analyze Scenarios 3 and 4, and examine how the number and the position of direct base station connections affect time delays.

**Figure 6:**
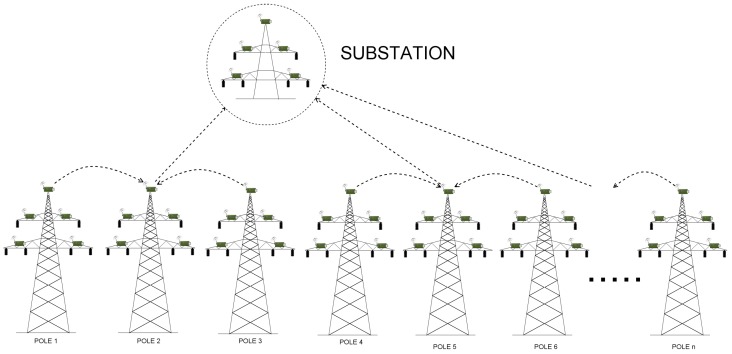
WSN architecture deployed on the example transmission grid, where η=2.

**Figure 7:**
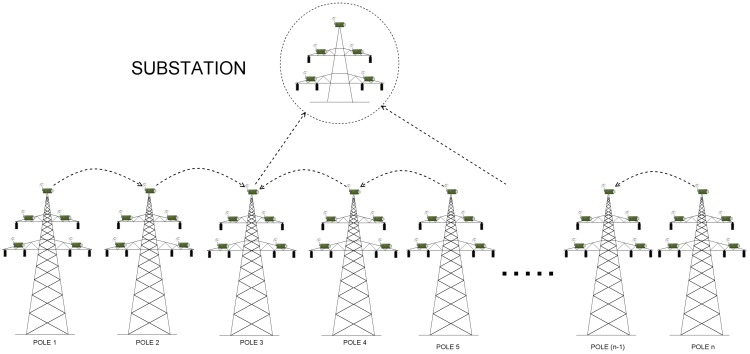
WSN architecture deployed on the example transmission grid, where η=3.

Consider, for instance, Scenario 3, where the number of direct links is equal to 12. Here, relay from node 1 and 3 both send their information to node 2, which has the direct link and will be able to send the data to the substation. Then, nodes from pole 4 and 6 send data to node 5 (which is responsible for communicating with the base station), nodes 7 and 9 send the data to relay on pole 8, which communicates with one of the available substations and can transmit the data using its direct link), and so forth (Figure 6 and Figure 7).

***Scenario 5*** The last but not least, Scenario 5 examines the performance and time delays when there is only one substation and the measured data from every pole need to traverse the entire power line in order to reach the substation. From the performance point of view, it does not make a sense to consider the number of hops to be greater than 23—the half of the line. The scenario with η equal to 45 is considered only as an example, in order to show that the approach, where the number of hops is greater than the half the length of the line, is inefficient and should not be used.

Prepared scenarios, together with factors significant in our analyzes, are summarized in Table 3.

## 4. Transmission Grid Model in Quality of Protection Modeling Language

### 4.1. QoP-ML Overview

In the article [17] the QoP-ML was introduced. Proposed solution provides the modeling language for making abstraction of cryptographic protocols that puts emphasis on the details concerning the quality of protection. The intended use of QoP-ML is to represent the series of steps, which are described as a cryptographic protocol. The QoP-ML introduced the multilevel protocol analysis that extends the possibility of defining the state of the cryptographic protocol. Since approaches presented in the literature usually speak for an example of a model driven security, in the light of the available development methodologies, QoP-ML excellently fits in a design known as a Model-Driven Engineering. The Model-Driven Engineering (simply known as MDE) is meant to focus on the creation and utilization of the abstract representations of the knowledge that govern a particular domain, rather than on the computing, algorithmic or implementation concepts. Model-Driven Engineering approach is a broader concept than Model-Driven Architecture (MDA), or Model-Driven Security (MDS). MDE adds multiple modeling dimensions and the notion of a software engineering process. The various dimensions and their intersections together with a Domain-Specific Language (DSL) form a powerful framework capable of describing engineering and maintenance processes by defining the order in which models should be produced and how they are transformed into each other. Serving as a domain-specific language, QoP-ML is capable of expressing security models in a formalized, consistent and logical manner.

As is apparent from the above description, QoP-ML is a flexible, powerful approach to model complex IT environments. Therefore, we utilized it to prepare our case study and evaluate the quality of chosen security mechanisms using its supporting, automatic framework. In the following sections we present all the significant components of the language we utilized to create model for our scenario.

### 4.2. General Information

The structures used in the QoP-ML represent high level of abstraction which allows concentrating on the quality of protection analysis. The QoP-ML consists of processes, functions, message channels, variables, and Quality of Proection (QoP) metrics. Processes are global objects grouped into the main process, which represents the single computer (host). The process specifies behavior, functions represent a single operation or a group of operations, channels outline the environment in which the process is executed. The QoP metrics define the influence of functions and channels on the quality of protection. In [17] the syntax, semantics and algorithms of the QoP-ML are presented in greater detail.

### 4.3. Data Types

In the QoP-ML, an infinite set of variables is used for describing communication channels, processes and functions. The variables are used to store information about the system or specific process. The QoP-ML is an abstract modeling language, so there are no special data types, sizes or value ranges. The variables do not have to be declared before they are used. They are automatically declared when used for the first time. The scope of the variables declared inside the high hierarchy process (host) is global for all processes defined inside host.

### 4.4. Functions

The system behavior is changed by the functions, which modify the states of the variables and pass the objects by communication channels. During the function definition, one has to set the arguments of this function which describe two types of factors. The functional parameters, which are written in round brackets, are necessary for the execution of the function and the additional parameters which are written in square brackets and have an influence on the system’s quality of protection. The names of the arguments are unrestricted.

### 4.5. Equation Rules

Equation rules play an important role in the quality of protection protocol analysis. Equation rules for a specific protocol consist of a set of equations asserting the equality of function calls. For instance: the decryption of the encrypted data with the same key, is equal to the encrypted data.

### 4.6. Process Types

The processes are the main objects in the QoP-ML. The elements which describe the system behavior (functions, message passing) are grouped into processes. In the real system, the processes are executed and maintained by a single computer. In the QoP-ML the sets of processes are grouped into the higher hierarchy process named host. All of the variables used in the high hierarchy process (host) have a global scope for all processes which are grouped by the host. Normally, the variables used in the host process cannot be applied to the other high hierarchy process. This operation is possible only when the variable is sent by the communication channel.

### 4.7. Message Passing

The communication between processes is modeled by means of channels. Any type of data can be passed through the channels. The channels must be declared before the data is passed through. The data can be sent or received by the channels. The channels pass the message in the First In First Out (FIFO) order. When the channels are declared with the non-zero buffer size, the communication is asynchronous. The buffer size equal to zero stands for the synchronous communication. In synchronous communication, the sender transmits the data through the synchronous channel only if the receiver listens on this channel. When the size of the buffer channel equals to at least 1, then the message can be sent through this channel even if no one is listening on this channel. This message will be transmitted to the receiver when the listening process in this channel is executed.

### 4.8. Algorithms

The main reason of introducing the algorithm structure was the need of specifying a series of well-defined successive states, that would let one to precisely define a non-linear and variable execution time of the given function. The inspiration to create this structure was an attempt to determine the packet’s transmission time, which is not directly proportional to the size of the transmitted data. Algorithms were introduced along with the advanced network analysis module in [18]. Algorithm definition is similar to the definition of the function. It is enclosed inside the algorithms structure, and starts with the alg keyword, followed by the algorithm name. Each algorithm has one parameter which is a message being sent in the case of a communication step or a function call expression in the case of an operation in process. Implementing an algorithm in QoP-ML, it is possible to use arithmetic operations, constructions known from the C language, such as conditional statements (if), loops (while), and two pre-defined functions: quality and size. The built-in, quality function can be used only in algorithms, which calculate communication time step and return the quality of the link between the sender and the receiver (that is, the *q* parameter). On the other hand, the size function is used to determine the size of the algorithm’s parameter, which can be a function call or a sent message (or one of its elements, accessed by index). Inside the algorithms structure, one can define as many algorithms as needed.

### 4.9. Security Metrics

The system behavior, which is formally described by the cryptographic protocol, can be modeled by the proposed QoP-ML. One of the main aims of this language is to abstract the quality of protection of a particular version of the analysed cryptographic protocol. In the QoP-ML, the influence of the system protection is represented by the means of functions. During the function declaration, the quality of protection parameters are defined and details about this function are described. These factors do not influence the flow of the protocol, but they are crucial for the quality of protection analysis. During that analysis, the function’s quality of protection (QoP) parameters are combined with the next structure of QoP-ML, the security metrics. In this structure, one can abstract the functions’ time performance, their influence on the security attributes required for the cryptographic protocol or other important factors during the QoP analysis.

### 4.10. Model Description

Analyzing the transmission line monitoring scheme, we utilized the QoP-ML to prepare a functional model of the considered segment of smart grid environment. We gathered and used real hardware security metrics and developed appropriate scenarios in order to examine how different factors influence transmission time delays, lifetime of sensor devices, confidentiality, integrity, and availability of the data traversing transmission grid environments. In this section, we briefly discuss all the elements we prepared to create the transmission grid models, and analyze the results we gathered using the Automated Quality of Protection Analysis (AQoPA) tool [19]. (The AQoPA tool performs the automatic evaluation and optimization of complex system models which are created in QoP-ML.) In this article, describing our model, we considered only the scenario where η=2. (However, remaining scenarios are designed analogically, so they can be understood without further explanation.) Both the models and the AQoPA tool can be downloaded from the QoP-ML’s project webpage [20].

In QoP-ML, in order to perform a simulation, one needs to prepare and implement 3 basic elements, namely: the model itself, hardware security metrics and miscellaneous scenarios of the considered environment (which are also called versions). For more information about QoP-ML, its syntax, semantics, algorithms and example usages, please refer to [17,21].

Before we present implementation details, let us describe the model in general. We have 4 types of communicating hosts: manager, sink, sensor and a substation (in our model referred to as the base station). While there is no manager in a real life deployment of the considered scenario, its abstraction in QoP-ML needs the manager to handle proper packet flows. Manager stores lists of sinks and sensors, and knows which sensor is assigned to which sink. Its main role is to send control messages to sink nodes, in order to give them instructions from which sensors they should collect data from, or when it is the best time for performing data transmission to the substation. Sink nodes receive a list of its sensors from manager, generate some parameters, send them to assigned motes and wait for the collected data. After the data is collected, sensors send it to their relay node. When the relay node receives a message from each of four sensors and the manager, it immediately starts the routing to the substation. Communication ends when the base station receives packets from all the sinks that connect directly.

In Listing 1 we present functions prepared for the transmission grid model. Lines 3–7 contain declaration of functions, which are used by the manager to, for instance, handle the division of sensors to proper sinks. Functions representing the type of the message traversing the transmission grid environment are defined in lines 8–14. Symmetric encryption and symmetric decryption functions (lines 16–17) take two functional arguments, the data to be encrypted/decrypted (data) and the key used for encryption/decryption (K). Besides functional arguments, s_enc and s_dec take two additional, QoP parameters: the algorithm used for encryption/decryption, and the size of the key in bytes (key_size). Remaining functions (lines 19–21) refer to data collection, and are fairly self-explanatory.

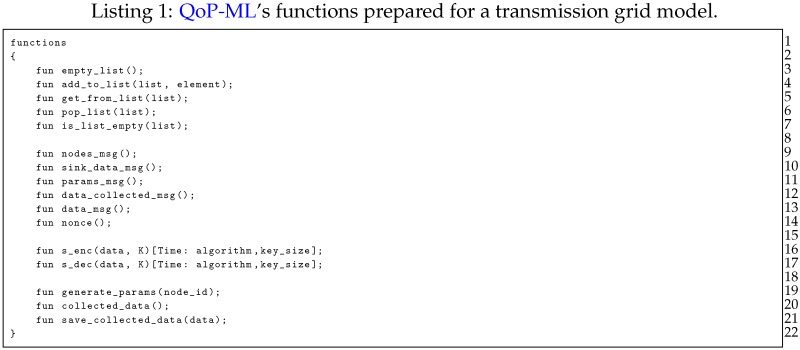



Equational rules, needed by list operations and symmetric cryptographic functions, are defined in Listing 2, and are simply used to assess the equality of function calls and to reduce complex function calls. For instance, equation in line 7, states that the symmetric decryption of symmetrically encrypted data with the same key returns the encrypted data.

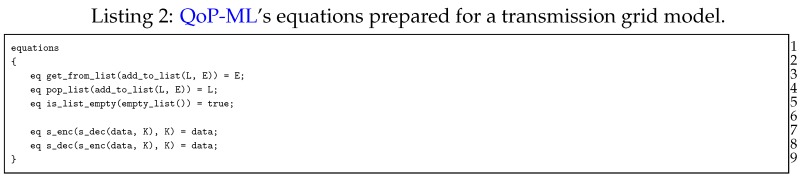



Secure communication between sinks all over transmission line is a crucial element in our simulation: communicating hosts need a medium to exchange messages. For this reason, implementing considered environment, we defined two communication channels (Listing 3): ch_WSN and ch_MGNT. Due to the fact that the manager is only a helper host, and its role is to handle packet flows, the manager uses ch_MGNT channel for sending control information (the one, which has connection quality equal to 1 (the lower the *q* value, the better the connection quality), and sending time equal to 0 ms). On the other hand, ch_WSN is responsible for transferring all the data traversing a transmission grid. Exact channel characteristics will be discussed while presenting the versions structure.

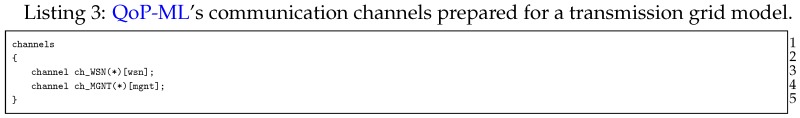



Moving on to the next point, let us now discuss hosts, which take part in a transmission grid communication, starting with the BaseStation host (Listing 4). The role of the base station is quite simple: in an infinite loop (lines 7–18), it waits for the data from sinks scattered over transmission line (lines 9–10), decrypts the data (only when it was encrypted (lines 12–15)), and saves it for further processing (line 17). Next, we have the Sensor host (Listing 5), responsible mainly for data collection. The very first instruction of the Sensor host is the generation of the symmetric key (line 3), which can be further used for data encryption (line 16). Sensor waits for parameters send by sink (lines 7–9), in which there is an identifier (ID) of the device, which acts as the relay node for this specific sensor. Then, the sensor performs a measurement to gather the data (lines 11–12) and optionally encrypts it (lines 14–17).

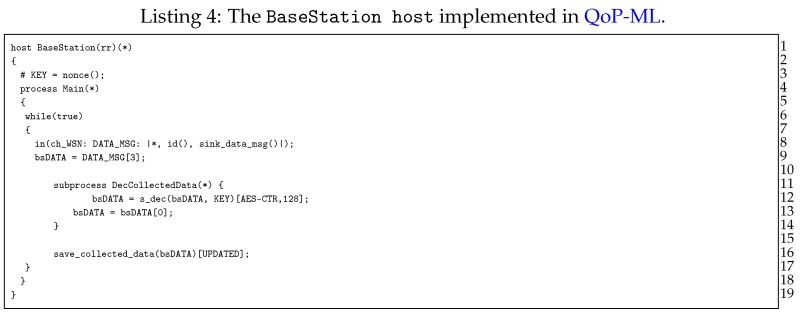

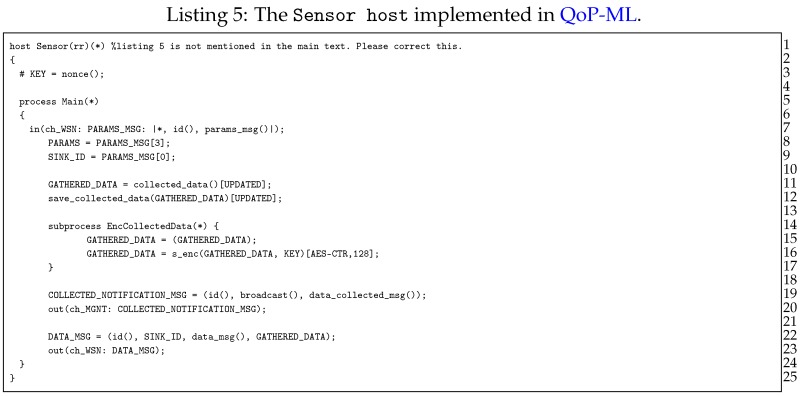





After the data is collected, sensor notifies everyone (in this case the manager, since it waits for the message with the data_collected_msg() type, which indicates that the data is gathered, and can be collected by appropriate relay nodes (lines 19–20). When the sink node is ready to receive the data, sensor simply creates a message and sends it to its relay node, using previously obtained SINK_ID (lines 22–23).

The most essential part in our simulation is the routing of the data obtained from all sensors towards the base station (substation actually). Sink devices (Listing 6) are those responsible for data gathering and transmission.

As it can be seen in Listing 6, sink consists of 3 processes, namely Main, WaitForData and HopByHopComm, where each of them is in charge of different operations. The Main process (lines 6–22) waits for data from Manager, in order to receive the list of sensors, from which sink needs to gather the data (lines 8–9). After receiving the list of sensors, sink generates parameters and sends them to sensor devices (lines 13–20). The actual data gathering takes place in WaitForData process (lines 24–43), in which sink node waits for data gathered by sensors (lines 31–32). If the data was encrypted, sink decrypts it (lines 34–37), saves (line 39) and adds it to the list of the collected packets (line 40). The last, HopByHopComm process, is responsible for the actual data transmission towards the base station (lines 44–65). Its role is to receive the initial data from Manager or another Sink within its cluster (lines 47–48). Then, sink decrypts the data (but only if it was encrypted (lines 50–53)), adds the data to the list of the gathered packets containing measurement information (line 55) and performs the routing algorithm, in order to find the ID of the next sink on a path to the base station (line 56). Remaining instructions concern optional encryption (lines 58–61) of the collected data and the process of sending it to another sink, towards the base station. HopByHopComm process ends when packets from all the cluster heads reach the base station.

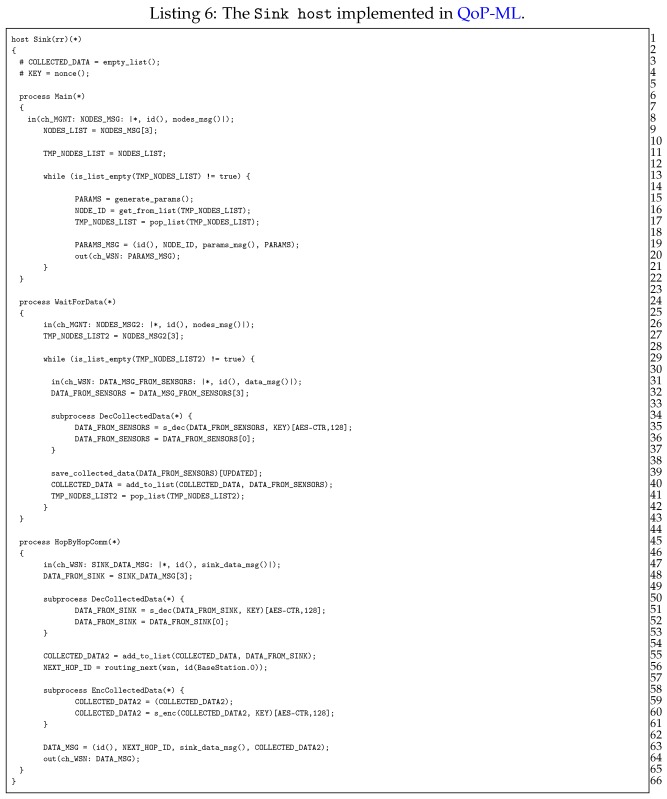



Last but not least, the Manager, represents a host, which is not available (and not needed) in a real-life deployment. As brought up earlier, its role is to work only as a helper host, which manages packet flows in our simulation. The Manager host consists of two processes, namely PrepareMessages and Main processes. The PrepareMessages process is responsible for creating lists of sensors, which belong to appropriate sinks along the transmission line (lines 7–39). Another function of this process is to maintain the list of sinks (lines 41–64), to which Manager has to send the initial message, which then starts the data gathering and routing process. Acting as a fundamental managing process, Main process’ first job is to inform each sink along the transmission line, from which sensors they can collect data from. Manager does that by sending the nodes_msg() type message (lines 69–84) to every sink. After sinks notification, Manager copies the list of all the available sinks (line 86) and collects information indicating end of data gathering process from TelosB sensors (lines 88–92). When all nodes_msg() messages are gathered, Manager prepares an empty list, which will be further send to each cluster, starting data routing process (being optionally encrypted before (lines 95–99)). Next, a list of all sinks (which are initiators of data routing within a cluster), is copied to a temporary variable (line 101). The last activity of the Manager host is to send the empty message to all the sinks, which initiate communication towards the base station within a given cluster (lines 103–109). Manager simply takes an ID of the sink from previously prepared list (line 105), removes it from mentioned list (line 106) and prepares a message (an empty list, actually, line 107), which is next send to each sink, one by one, until the list ends (line 108).

In order to determine the transmission time of the packets being sent through the transmission grid, we implemented an algorithm, which calculates the transmission time between two sensors (Listing 7). The transmission time of a single packet is equal to constant 18 ms plus 0.12 ms per each byte. The while loop is used to handle messages with the payload larger than 110 bytes, which is the maximum payload size in ZigBee (assuming that header has 17 bytes size; the maximum size of packet is 127 bytes) [22]. When the maximum size is exceeded, payload is divided into many packets with 110 bytes each.

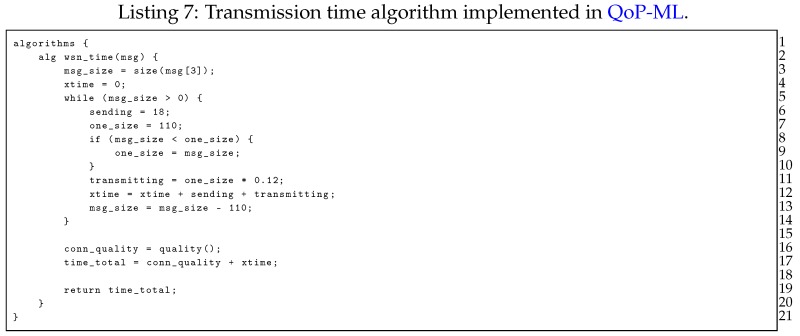

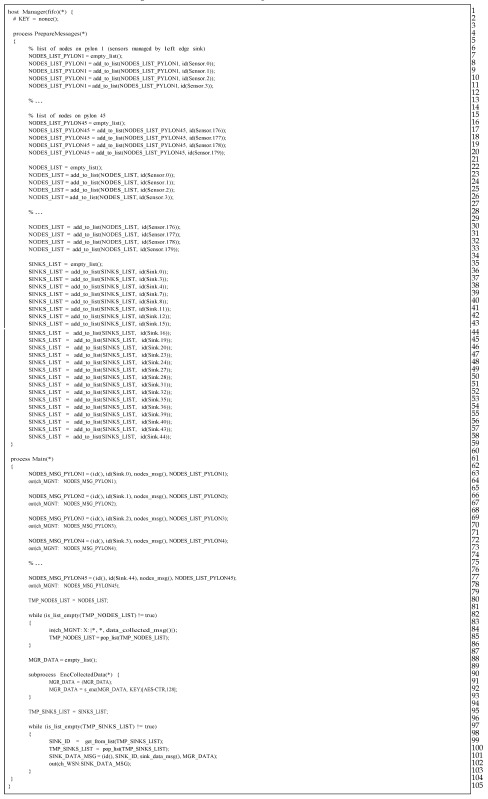





Another important step in QoP-ML’s modeling is the gathering of security metrics. This process can be fully (or partly) automated, using the Crypto Metrics Tool [23]. Hardware security metrics (like, for instance, time taken by encryption) can be gathered by the tool, while remaining ones can be added by hand. In Listing 9 one can see metrics obtained from TelosB devices.

Let us focus on, for instance, lines 26 and 27 from Listing 9. Here, the details about the encryption process performed by TelosB devices are defined. As we can see from the metric’s header (line 26), we have some information about the considered operation type, utilized algorithm, the size of the encryption key, the time taken by the encryption itself (in ms), and finally, the size of the encrypted message (in bytes). Exact values, corresponding to elements available in the metric’s header, are given as follows.

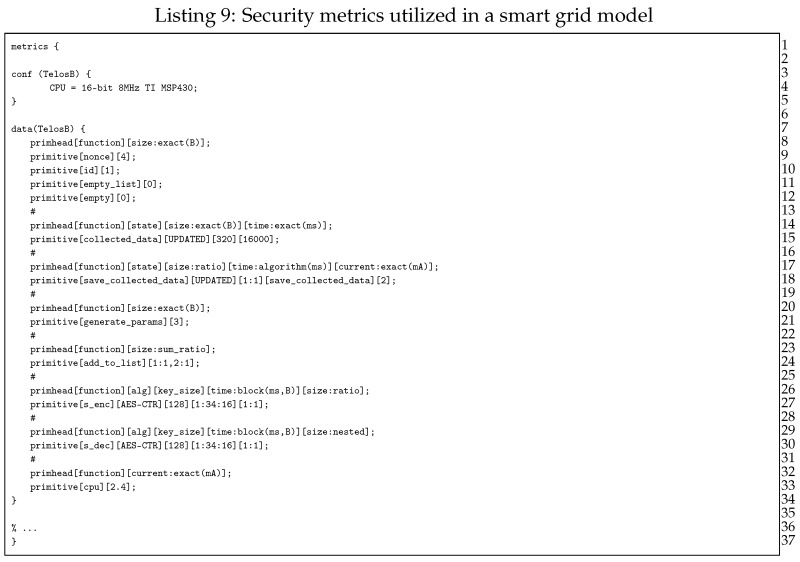



The operation type (function name) is s_enc, alg refers to the utilized encryption algorithm, which, in this case is AES-CTR. The key_size is equal to 128 bytes, while the remaining parts concern time and output size calculations (line 27). Gathered security metrics are utilized during the simulation process to take into account the influence of the hardware specifications on performed operations. For more information about security metrics itself, its syntax, semantics and usage, please refer to [17,19,21].

Building the versions structure is considered the final step before the actual simulation. Preparing multiple versions, one is capable of defining different sequences of events, without a need to modify the model. In the considered transmission grid analysis, we examine only two main scenario types: with (version Enc_Updated) and without (version NoEnc_Updated) packet encryption (Listing 10). Analyzed scenarios differ only in hosts’ instruction start-up process: the Enc_Updated version, except running base processes, additionally starts up sub-processes responsible for cryptographic operations. Let us now discuss the role of each instruction, as they are used in both version structures.

Lines from 5 to 8 (the set instructions) link hosts with appropriate security metrics, so that the operations performed by those hosts make use of suitable values (bear in mind that, for instance, encryption time can be different for TelosB and MicaZ motes). The following lines (10–27) are those which differ between versions: in NoEnc_Updated version, run instructions start only basic processes, without even knowing about sub-processes, while Enc_Updated (lines 93–110) additionally executes commands responsible for packet encryption. Another important role of the run instruction is the possibility to define how many host instances one can start. As shown in Listing 10, our WSN consist of 45 MicaZ sinks, 180 TelosB sensor devices, a base station, and the manager. Communication between utilized devices is defined inside the communication structure, between 29th and 84th line. Here, communication channel characteristics for each medium (such as the time taken by sending and receiving packets or current values) are given (lines 31–34).

The topology of the utilized WSN is specified with the help of the topology structure (lines 36–63). Every connection defined in topology structure consists of communicating hosts, and an arrow which expresses the actual packet flow. Consider, for instance, the 51st line, where one can see two communicating sites: sink and base station. As can be seen, second from 45 sinks sends the packet straight to the base station (as indicated by an arrowhead, which points to the right, that is, from sink to base station). The topology structure is a very flexible and adaptable part of QoP-ML, since it allows defining details of each connection separately, taking into account its key characteristics. For more information about versions structure, refer to [17,19,21].

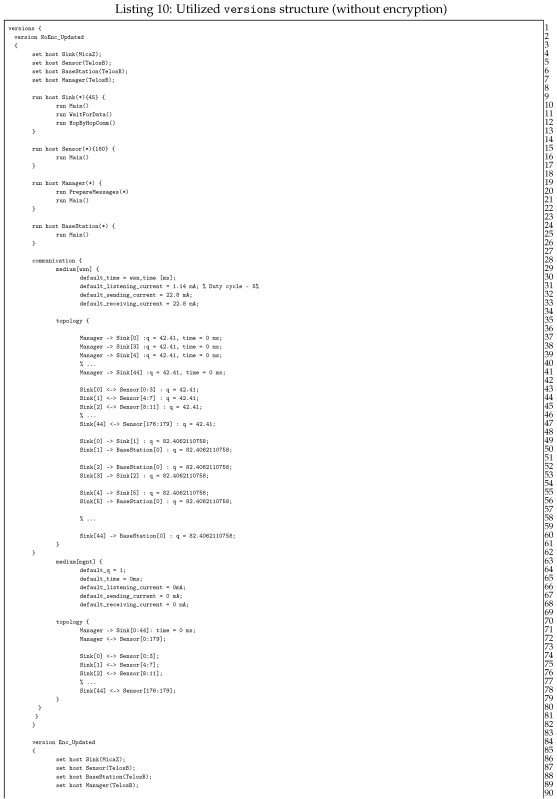

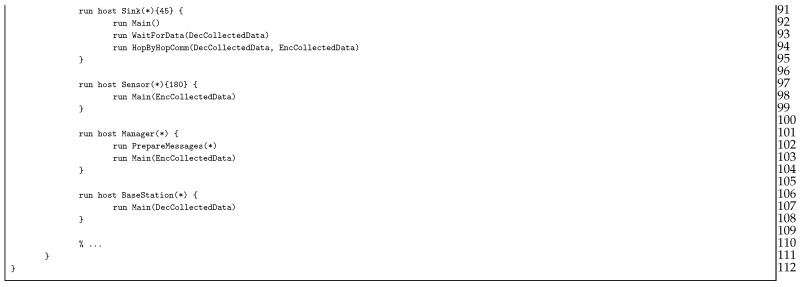





## 5. Results Analysis and Discussion

In this section we study the results gathered for several different scenarios, including variation in the number of groups, direct connections to the substation, routing algorithms and security mechanisms. We compare the results of our proposed formulation and evaluate time delays of the network, including the cases of constrained cellular coverage. The simulation results of various implementation scenarios of WSNs and overhead smart grid networks aim at highlighting their performance and examining how inherent and imposed factors influence the maximum delay time of the different arrangement scenarios. The below analysis concerns only the results obtained for the transmission line, where the distance between two adjacent poles is equal to 100 m. Remaining results (for distances equal to 50, 200 and 300 m) are summarized in the Appendix section, in Table A6, Table A7, Table A8, Table A9, Table A10, Table A11, Table A12, Table A13, Table A14 and Table A15. All the system parameters as well as their default values, that are involved in the following analysis, are reported in Table 3. These values help towards the establishment of realistic implementation scenarios. Note that all the assumptions of Section 3 are also satisfied.

Table 4 contains the results gathered for the first scenario (Scenarios 1a,b). In this scenario, we consider the transmission line to be divided into two sub-lines, where each of them has only one direct link to the substation (κ=2). Thus, the number of hops (intermediary relay nodes), obtained data should traverse to finally reach the substation, is equal to 22 for the left sub-line, and 23 for the right sub-line (η=22, η=23). The size of the measurement data gathered by each sensor at every tower is the same, and is equal to 80 bytes. It takes about 4000 ms for TelosB to transmit it to the relay node. Because there are 4 measurement sensors at each pole, the size of the data received by the relay node is 320 bytes, and the time taken for its transmission is calculated using the algorithm available in QoP-ML model.

As we look at the results in Table 4, we can see that the time needed by sink to send the measurement data to the neighboring relay node increases along with the number of hops, and it is always the longest for the last node on line (sink 22 and sink 45). Furthermore, the difference between transmitting encrypted and unencrypted data grows rapidly as well. Additionally, the results in Table 4 indicate that this linear network model suffers from an imbalance of workload. The relays located closer to the substations have to handle a lot more traffic than those sitting farther away on the line. Considering the topological constraints posed by the transmission lines, the low bandwidth, low data rate wireless nodes fail to transmit huge amounts of data in a multi-hop manner.

As a consequence of analyzing results gathered for Scenario 1, it was necessary to identify a more efficient way of delivering collected data to substations. In order to balance the workload of transmitting sinks, we established Scenario 2, where each relay on every pole along the transmission line has a direct connection to the substation. The size of transmitted data and transmission times are the same as in Scenario 1. Table 5 consists of the results gathered for this Scenario 2a,b. As is evident from Table 5, by dividing all nodes into κ=45 stand-alone groups, we managed to solve the issue of imbalanced workload: the time needed by each sink is almost equal, no matter if we consider the first, or the last relay node on line. Moreover, the difference in transmission times between encrypted and unencrypted traffic is almost constant—it does not change as severely as in Scenario 1. Although this approach has many advantages (balanced workload, the constant time difference between various security options), there exist some drawbacks as well. One of them is cost—deploying cellular transceivers on each tower is a very expensive solution. While such a network can provide extremely low latency data transmission, this model is highly cost inefficient as it incurs huge installation and subscription costs. This is why this solution can not be used in real-life deployments.

Because the problem of finding optimal locations of cellular transceivers is such a complex task, that two previous scenarios failed to succeed, we proposed to consider another approach (Scenarios 3a,b, 4a,b). Since the idea of splitting relay nodes into groups succeeded in terms of workload balancing, we decided to adhere to established rules. However, we modified them such that we divided relay nodes into less than 45 groups, in order to keep the balance and additionally decrease cost. As we take a look at the results in Table 6 and Table 7, we see that the performance of each sink is still stable, even with the smaller number of groups (κ=12 for Scenario 3, and κ=8 for Scenario 4). Also here, the time increases along with the number of hops, but the growth is not so rapid as in the case of Scenario 1. Moreover, the difference between secure and insecure version remained more or less constant.

As the main role of wireless sensors in our research is to monitor electric power systems and react in a timely manner to possible power system disturbances, the above examinations can be a great help. Performing analyzes with QoP-ML, we are able to choose the appropriate network architecture, routing algorithms, and security mechanisms for reliable and accurate monitoring of specific power system actions and disruptions. As previously stated, in power grid systems, 4 main disturbances can be distinguished, namely: wave, electromagnetic, electromechanical and thermodynamic disruptions. Such classification of power grid disturbances is related to the time range, within the given event should be spotted and reported. In order to let smart grid operators react to occurred power grid disturbance in an appropriate way, the time between the event occurrence and the time it has been reported by monitoring wireless sensors should fall within a suitable range (Figure 1, Table 1). Simulation results helped us to decide, which of the proposed scenarios should be used for monitoring given power grid disturbances.

**Figure 8:**
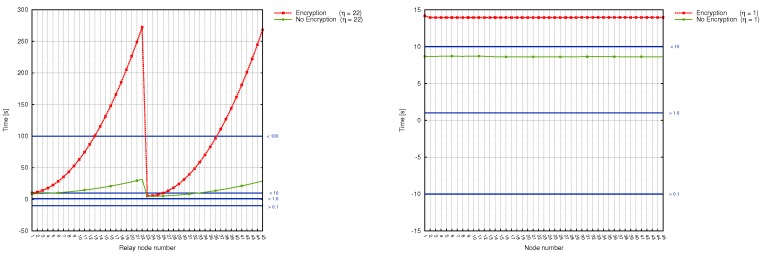
Scenarioss 1a,b, 2a,b: correlation between the sink number, time and power grid disturbance time frames (encryption/no encryption).

**Figure 9:**
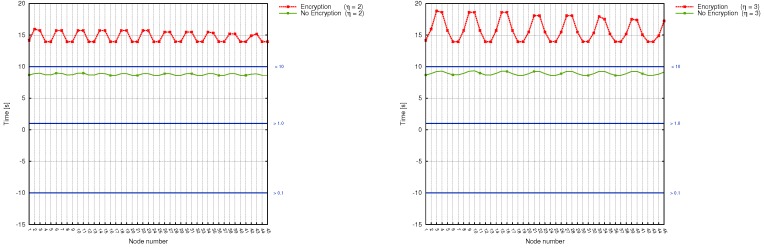
Scenarioss 3a,b, 4a,b: correlation between the sink number, time and power grid disturbance time frames (encryption/no encryption).

**Figure 10:**
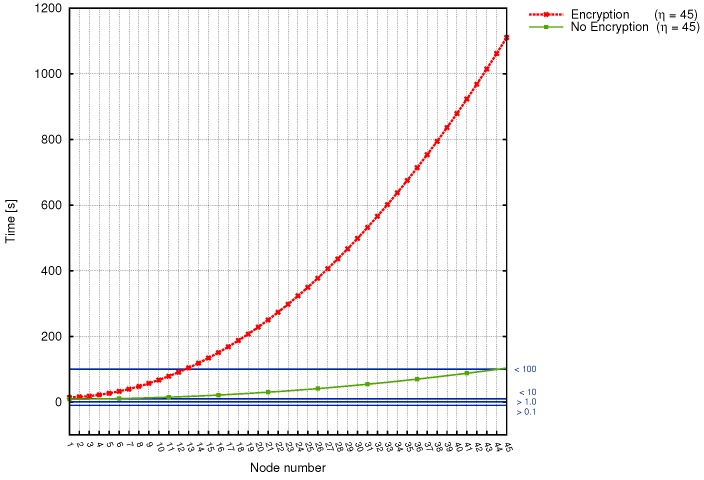
Scenarios 5a,b: correlation between the sink number, time and power grid disturbance time frames (encryption/no encryption).

We made a correlation between the number of relay nodes on the way to substation, the time taken by sinks to deliver measured data together with power grid disturbance time frames (Figure 8, Figure 9 and Figure 10). Additionally, in Table 9 we gathered existing power systems transients and using simulation results, determined which from prepared scenarios can be used for monitoring specific transmission grid phenomena. It turned out, that different scenarios are capable of monitoring different phenomena, and in order to achieve the most reliable, useful results and react in a timely fashion to power grid disturbances, a profound analysis of the transmission grid architecture should be accomplished. As it is evident from Table 9, wireless sensors seem to be not the best choice when it comes to wave and electromagnetic phenomena monitoring. (This applies to Scenarios 1, 2, 3 and 4. For this type of power grid disturbances, one needs a faster solution (for instance, the optical fiber). When we take a look at the time requirements of electromechanical and thermodynamic phenomena monitoring, it is clear that all prepared scenarios can handle this task. However, from the performance and economic point of view, it is preferable to choose Scenario 4 instead of Scenario 3 or 2 (since it requires fewer direct connections to the substation, which directly influence the total cost of WSN deployment and is characterized by a better capability for load balancing). Although we did not consider the scenario, where the data needs to traverse all sinks along the power transmission line in order to reach the base station as a promising WSN architecture for smart grids from the beginning, the above analysis confirmed this thesis even more.

The issues raised by the first scenario can be easily noticed in Figure 8 (left graph), which contains time results for the left-subline (nodes of numbers from 1 to 22) and right sub-line (nodes from 23 to 45). The problem of workload imbalance can be observed for nodes, which reside closer (in terms of distance) to the substation (such as, for instance, node number 22 and 45). The time needed by them to serve network requests is equal to about 4.5 min (while the nodes which reside at the beginning of each sub-line need less than a minute to perform their task), and, what is more important, is not constant for each wireless node.

Scenarios, where wireless sensors are divided into groups, bring a significant improvement, as evidenced by Figure 8 and Figure 9. Here, the workload is more or less stable and decreases for each sensor along with η. Time delays are as well not so big as in the case of Scenario 1 and range between a couple of seconds (both for encrypted and unencrypted version). They, however, increase together with the number of hops inside the group. The most stable and delay-aware from the above 3 , is the second scenario. At the same time, however, it is the most expensive one to deploy.

On the other hand, the comparison of transmission times of encrypted and unencrypted data for Scenarios 5a,b can be observed in Figure 10. As it can be seen, the difference between transmission time is significant here. Gathered results clearly indicate, that when the encryption is used, the transmission time grows rapidly, causing greater time delays. On the other hand, when we consider the non-encrypted data transmission, it is evident that this approach performs better in terms of minimizing time delays. For the transmission line consisting of 45 electric poles, the longest transmission time is equal to, approximately 18 min.

Analyzing the results gathered for different scenarios, it is worth examining how the distance between the poles affects the transmission time. As it can be observed from the results in Table A1, Table A2, Table A3, Table A4, Table A5, Table A6, Table A7, Table A8, Table A9, Table A10, Table A11, Table A12, Table A13, Table A14 and Table A15Table A15, when the distance between the poles changes, transmission time delay slightly increases. Consider, for instance, nodes 1, 22 and 45. The time difference between first scenarios for different distances and node 1 is equal to about one tenth of a second. (The same is true for Scenarios 2–5). Node 22, located in the middle of the transmission line, is as well characterized by negligible differences in transmission time when considering different distances. For node 22, only Scenarios 1 and 5 have slightly larger changes in transmission time when comparing the delays on given distances. Identical situation applies to sensor 45. The time difference between different distances exists in every scenario. However, it is so small, that it is almost imperceptible.

### 5.1. Security Considerations

As mentioned in the *Scenarios* section, each of the proposed scenarios has two versions (Table 3): the secure version (always referred to as the (b) scenario, where, among others, the encryption is used) and the second, insecure version, where there are no security mechanisms at all (the (a) scenarios). For instance, scenario 1a, 2a or 5a are insecure, while scenarios labelled with (b) (1b, 2b, ...) are secure versions of the proposed solution. In Table 10 we gathered some fundamental security concepts (such as confidentiality, integrity, authentication, freshness and availability) and evaluated proposed scenarios in terms of these security attributes, in order to find out which of these scenarios provide the selected security features.

Confidentiality of data in a sensor network is achievable only if those with access to network are authorized to do so. Under no circumstances should sensor readings leak outside the network. The standard approach for preventing this from happening is to use encryption. The secure version of the introduced protocol uses encryption in order to protect the information from disclosure to unauthorized parties. In the case of the insecure version, because of the lack of the encryption, we can not protect information confidentiality.

When it comes to integrity, both solutions can not guarantee it. However, if data integrity is a specific concern, one should use a cryptographic hash function but combined with an an encryption algorithm. Symmetric ciphers (such as the Advanced Encryption Standard (AES) algorithm working in counter mode (CTR) mode utilized in our secure scenarios) do not (by themselves) provide integrity, because they do not detect malicious or accidental modifications to data. In order to provide integrity, the solution is to wrap the data inside packages with the data that can be used to validate the integrity of the package, typically a hash-based checksum.

The process of authentication is very important in preserving network data integrity and preventing unauthorized access to the network. Without the authenticating mechanisms, an attacker can easily access the network and inject dangerous messages without the receivers of the data knowing that the data being used originates from malicious source. In the secure version of the protocol, the authentication is assured in a way that we use individual encryption keys for every node taking part in communication process. Encryption keys are hard-coded in every device. Insecure scenarios do not provide authentication.

The choice of good security mechanism for WSNs depends on network application and environmental conditions. In a transmission grid environment, where wireless sensors are deployed on an electric pole, the availability should not be a big concern. Despite the batteries, sensors can be equipped with a solution, that could power up sensors, using the energy from the electric line. In the case of the disturbance in power supply from the electric line, wireless nodes can use the backup source of energy (the batteries). However, this solution brings an additional risk: a new kind of the Distributed Denial of Service (DDoS) attack, the delayed distributed denial of service, referred to as the Delayed Distributed Denial of Service (DDDoS) attack [24]. The DDDoS attack is especially dangerous for WSNs, where the energy is one of the constrained resources: it can decrease the lifetime of the sink node by slowly and imperceptibly consuming its valuable power, leading to total exhaustion of energy resources and being unnoticed by the traditional DDoS defense mechanisms.

## 6. Conclusions

Wireless sensors, intelligent substations and communication devices provide real-time information on system health so that smart grid operators can pro-actively prevent many issues. Using real-time information from wireless sensors and automated controls to detect and respond to system problems, a smart grid can automatically avoid or mitigate power outages, power quality problems, and service disruptions. However, all of the existing communication links in a smart grid environment introduce vulnerabilities, especially if they can be accessed over by a wireless medium. Thus, serious security analysis is needed to ensure that implemented solutions are truly adequate. In this paper, we proposed a reconfigurable network model and examined it in terms of security and time delays. Our objective was to minimize the time delays, depending on the number of sinks, clusters and confidentiality of the transmission. We did, however, take into account neither energy constraints, nor cost of deployment. Prior to simulations, we presented the general formulation of network architectures, scenarios and time delay problem. First, we analyzed the linear network model and came to conclusion that it suffers from the time delay issue and imbalance of workload. Nevertheless, we proved that its performance can be improved by careful choice of the position of direct wireless links. Later, after we investigated the first model, we utilized an optimization approach in order to minimize the maximum time delay. By examining different scenarios, we confirmed that the improvement made by the proposed solution is significant: Minimizing time delays, we managed to decrease the cost of network deployment ensuring secure communication at the same time.

The approach proposed in this paper defines an efficient, low cost, and easily configurable network model. It is generic and encompasses a variety in several factors. Its flexibility and the ease of reconfigurability lets us examine miscellaneous network configurations and decide which of them will satisfy our needs best. We believe that the result of this paper can provide invaluable guidances for smart grid developers, and help them design future smart grid to be well balanced in terms of performance, cost and time delays.

## Figures and Tables

**Table 1:** Classification of power system transients and their time scales.

Phenomena	Time Frame Seconds (s)	Disturbances and/or Actions
Wave	$10^{-7}$ – $10^{-2}$	Switching transients during opening and closing power line circuit breakers or caused by lighting strike to the transmission line
Electromagnetic	$10^{-4}$ – $10^{0}$	Synchronous or sub-synchronous resonances during generator or electric motor switching to the grid
Electromechanical	$10^{-1}$ – $10^{2}$	Synchronous generator shaft swings caused by short-circuits or power balance disturbance
Thermodynamical	$10^{1}$ – $10^{5}$	Boiler regulatory actions in thermal power plants, secondary frequancy control, heating and cooling transmission line wires due to weather changes or disturbances

**Table 2:** Mica2 and TelosB hardware specification.

Characteristic	TelosB [15]	Mica2 [16]
Current Draw (sleep mode)	1.8 mA	8 mA
Current Draw (active mode)	5.1μA	<1μA
RF power	-24–0 dBm	-20–10 dB
Receive Sensitivity	-90 dBm (min), -94 dBm (typ)	-101 dBm
Outdoor Range	75–100 m	305 m
Frequency band	2400–2483.5 MHz	433 MHz
Data Rate	250 kbps	38.4 Kbaud
Communication Protocol	IEEE 802.15.4/ZigBee	IEEE 802.15.4/ZigBee
Cost	$182	$152

**Table 3:** Scenarios prepared for our case study.

Scenario	Number of Direct Links to Substation (κ)	Number of Hops (η)	Routing Type	Security Mechanisms
1(a)	2	22–23	hop by hop	No Encryption
1(b)	2	22–23	hop by hop	Encryption (AES-CTR, 128)
2(a)	45	1	direct link	No Encryption
2(b)	45	1	direct link	Encryption (AES-CTR, 128)
3(a)	12	2	central point	No Encryption
3(b)	12	2	central point	Encryption (AES-CTR, 128)
4(a)	8	3	central point	No Encryption
4(b)	8	3	central point	Encryption (AES-CTR, 128)
5(a)	1	45	central point	No Encryption
5(b)	1	45	central point	Encryption (AES-CTR, 128)

**Table 4:** Scenario 1: transmission times (in s) for each sink all over the transmission line. (Distance between poles is equal to 100 m, κ=2, η=22,η=23, NE means no encryption, E means encryption.)

Sink	Time${}_{\mathrm{NE}}$	Time${}_{E}$	Sink	Time${}_{\mathrm{NE}}$	Time${}_{E}$	Sink	Time${}_{\mathrm{NE}}$	Time${}_{E}$	Sink	Time${}_{\mathrm{NE}}$	Time${}_{E}$	Sink	Time${}_{\mathrm{NE}}$	Time${}_{E}$
1	8.72	9.97	11	14.6	74.5	21	29.57	248.91	31	8.36	39.4	41	21.29	180.85
2	8.9	11.48	12	15.69	87.0	22	31.58	272.4	32	9.25	48.6	42	23.09	201.04
3	9.16	14.08	13	16.86	100.61	23	4.61	5.41	33	10.23	58.91	43	24.97	222.33
4	9.5	17.78	14	18.12	115.29	24	4.75	5.93	34	11.3	70.31	44	26.95	244.72
5	9.95	22.58	15	19.48	131.08	25	4.98	7.3	35	12.46	82.81	45	29.02	268.21
6	10.5	28.49	16	20.93	147.97	26	5.32	9.9	36	13.72	96.42	46		
7	11.13	35.49	17	22.48	165.95	27	5.74	13.6	37	15.05	111.1	47		
8	11.86	43.59	18	24.11	185.04	28	6.26	18.4	38	16.47	126.89	48		
9	12.68	52.79	19	25.84	205.23	29	6.87	24.3	39	17.99	143.78	49		
10	13.6	63.09	20	27.66	226.52	30	7.57	31.3	40	19.59	161.77	50		

**Table 5:** Scenario 2: transmission times (in s) for each sink all over the transmission line. (Distance between poles is equal to 100 m, κ=45, η=1, NE means no encryption, E means encryption.)

Sink	Time${}_{\mathrm{NE}}$	Time${}_{E}$	Sink	Time${}_{\mathrm{NE}}$	Time${}_{E}$	Sink	Time${}_{\mathrm{NE}}$	Time${}_{E}$	Sink	Time${}_{\mathrm{NE}}$	Time${}_{E}$	Sink	Time${}_{\mathrm{NE}}$	Time${}_{E}$
1	8.67	14.16	11	8.71	13.95	21	8.62	13.95	31	8.63	13.96	41	8.63	13.95
2	8.67	13.95	12	8.69	13.95	22	8.62	13.95	32	8.63	13.96	42	8.63	13.95
3	8.67	13.95	13	8.67	13.95	23	8.62	13.95	33	8.63	13.96	43	8.63	13.95
4	8.7	13.95	14	8.62	13.95	24	8.62	13.95	34	8.63	13.96	44	8.63	13.95
5	8.7	13.95	15	8.62	13.95	25	8.62	13.95	35	8.63	13.96	45	8.63	13.95
6	8.7	13.95	16	8.62	13.95	26	8.62	13.95	36	8.63	13.96	46		
7	8.7	13.95	17	8.62	13.95	27	8.62	13.95	37	8.63	13.96	47		
8	8.68	13.95	18	8.62	13.95	28	8.62	13.95	38	8.63	13.96	48		
9	8.71	13.95	19	8.62	13.95	29	8.62	13.95	39	8.63	13.95	49		
10	8.71	13.95	20	8.62	13.95	30	8.63	13.97	40	8.63	13.95	50		

**Table 6:** Scenario 3: transmission times (in s) for each sink all over the transmission line. (Distance between poles is equal to 100 m, κ=12, η=2, NE means no encryption, E means encryption.)

Sink	Time${}_{\mathrm{NE}}$	Time${}_{E}$	Sink	Time${}_{\mathrm{NE}}$	Time${}_{E}$	Sink	Time${}_{\mathrm{NE}}$	Time${}_{E}$	Sink	Time${}_{\mathrm{NE}}$	Time${}_{E}$	Sink	Time${}_{\mathrm{NE}}$	Time${}_{E}$
1	8.67	14.16	11	8.98	15.72	21	8.62	13.95	31	8.89	15.48	41	8.63	13.95
2	8.9	15.94	12	8.69	13.95	22	8.89	15.72	32	8.62	13.96	42	8.81	14.89
3	8.96	15.72	13	8.67	13.95	23	8.89	15.72	33	8.62	13.96	43	8.86	15.17
4	8.7	13.95	14	8.93	15.72	24	8.62	13.95	34	8.89	15.48	44	8.63	13.95
5	8.7	13.95	15	8.89	15.72	25	8.62	13.96	35	8.89	15.33	45	8.63	13.95
6	8.96	15.72	16	8.62	13.95	26	8.89	15.48	36	8.62	13.96	46		
7	8.93	15.72	17	8.62	13.95	27	8.89	15.48	37	8.62	13.95	47		
8	8.68	13.95	18	8.89	15.72	28	8.62	13.96	38	8.89	15.19	48		
9	8.71	13.95	19	8.89	15.72	29	8.62	13.96	39	8.89	15.19	49		
10	8.98	15.72	20	8.62	13.95	30	8.89	15.48	40	8.62	13.95	50		

**Table 7:** Scenario 4: transmission times (in s) for each sink all over the transmission line. (Distance between poles is equal to 100 m, κ=8, η=3, NE means no encryption, E means encryption.)

Sink	Time${}_{\mathrm{NE}}$	Time${}_{E}$	Sink	Time${}_{\mathrm{NE}}$	Time${}_{E}$	Sink	Time${}_{\mathrm{NE}}$	Time${}_{E}$	Sink	Time${}_{\mathrm{NE}}$	Time${}_{E}$	Sink	Time${}_{\mathrm{NE}}$	Time${}_{E}$
1	8.67	14.16	11	8.98	15.72	21	9.25	18.08	31	8.62	13.96	41	8.89	15.04
2	8.9	15.94	12	8.69	13.95	22	9.25	18.08	32	8.89	15.33	42	8.62	13.95
3	9.23	18.82	13	8.67	13.95	23	8.89	15.48	33	9.25	17.93	43	8.63	13.95
4	9.29	18.6	14	8.93	15.72	24	8.62	13.96	34	9.25	17.53	44	8.81	14.89
5	8.96	15.72	15	9.29	18.6	25	8.62	13.96	35	8.89	15.19	45	9.13	17.23
6	8.7	13.95	16	9.25	18.6	26	8.89	15.48	36	8.62	13.95	46		
7	8.7	13.95	17	8.89	15.72	27	9.25	18.08	37	8.62	13.95	47		
8	8.94	15.72	18	8.62	13.95	28	9.25	18.08	38	8.89	15.17	48		
9	9.27	18.6	19	8.62	13.96	29	8.89	15.48	39	9.25	17.51	49		
10	9.34	18.6	20	8.89	15.48	30	8.62	13.96	40	9.25	17.38	50		

**Table 8:** Scenario 5: transmission times (in s) for each sink all over the transmission line. (Distance between poles is equal to 100 m, κ=1, η=45, NE means no encryption, E means encryption.)

Sink	Time${}_{\mathrm{NE}}$	Time${}_{E}$	Sink	Time${}_{\mathrm{NE}}$	Time${}_{E}$	Sink	Time${}_{\mathrm{NE}}$	Time${}_{E}$	Sink	Time${}_{\mathrm{NE}}$	Time${}_{E}$	Sink	Time${}_{\mathrm{NE}}$	Time${}_{E}$
1	8.67	14.03	11	14.82	78.56	21	30.03	250.38	31	54.31	531.92	41	87.68	923.35
2	8.9	15.55	12	15.93	91.06	22	32.04	273.59	32	57.23	566.12	42	91.5	968.53
3	9.19	18.15	13	17.13	104.26	23	34.15	297.9	33	60.25	601.42	43	95.42	1014.81
4	9.58	21.85	14	18.43	118.68	24	36.35	323.31	34	63.35	637.81	44	99.43	1062.19
5	10.06	26.65	15	19.81	134.21	25	38.64	349.82	35	66.55	675.31	45	103.54	1110.68
6	10.63	32.55	16	21.29	150.83	26	41.03	377.43	36	69.84	713.91	46		
7	11.3	39.55	17	22.86	168.54	27	43.5	406.14	37	73.22	753.6	47		
8	12.05	47.65	18	24.52	187.35	28	46.07	435.94	38	76.7	794.4	48		
9	12.88	56.85	19	26.28	207.26	29	48.73	466.83	39	80.26	836.28	49		
10	13.81	67.16	20	28.11	228.27	30	51.49	498.83	40	83.92	879.27	50		

**Table 9:** Possibility of the usage of each scenario for different power system dynamics.

	*Phenomena*	Wave	Electromagnetic	Electromechanical	Thermodynamical
*Scenario*					
1(a)		−	−	✓	✓
1(b)		−	−	−	✓
2(a)		−	−	✓	✓
2(b)		−	−	✓	✓
3(a)		−	−	✓	✓
3(b)		−	−	✓	✓
4(a)		−	−	✓	✓
4(b)		−	−	✓	✓

**Table 10:** Security attributes assured in each of the proposed scenarios.

	Scenario	Secure	Insecure
Security Attribute			
Confidentiality		✓	−
Integrity		−	−
Authentication		✓	−
Availability		✓	✓

## Appendix A. Results Obtained for Remaining Distances

### Appendix A.1. Results for the Distance Equal to 50 m

**Table A1:** Scenario 1: transmission times (in s) for each sink all over the transmission line. (Distance between poles is equal to 50 m, κ=2, η=22,η=23, NE means no encryption, E means encryption.)

Sink	Time${}_{\mathrm{NE}}$	Time${}_{E}$	Sink	Time${}_{\mathrm{NE}}$	Time${}_{E}$	Sink	Time${}_{\mathrm{NE}}$	Time${}_{E}$	Sink	Time${}_{\mathrm{NE}}$	Time${}_{E}$	Sink	Time${}_{\mathrm{NE}}$	Time${}_{E}$
1	8.71	9.96	11	14.54	74.43	21	29.44	248.78	31	8.31	39.35	41	21.18	180.74
2	8.88	11.47	12	15.61	86.93	22	31.44	272.27	32	9.19	48.54	42	22.97	200.92
3	9.15	14.07	13	16.78	100.53	23	4.6	5.4	33	10.16	58.84	43	24.84	222.2
4	9.48	17.76	14	18.04	115.21	24	4.74	5.91	34	11.23	70.24	44	26.82	244.59
5	9.92	22.55	15	19.39	130.99	25	4.97	7.28	35	12.38	82.74	45	28.88	268.07
6	10.46	28.45	16	20.84	147.87	26	5.29	9.87	36	13.63	96.33	46		
7	11.09	35.45	17	22.37	165.85	27	5.71	13.57	37	14.96	111.01	47		
8	11.81	43.54	18	24.0	184.93	28	6.22	18.36	38	16.37	126.79	48		
9	12.63	52.74	19	25.72	205.12	29	6.82	24.26	39	17.88	143.68	49		
10	13.54	63.03	20	27.54	226.4	30	7.52	31.25	40	19.49	161.66	50		

**Table A2:** Scenario 2: transmission times (in s) for each sink all over the transmission line. (Distance between poles is equal to 50 m, κ=45, η=1, NE means no encryption, E means encryption.)

Sink	Time${}_{\mathrm{NE}}$	Time${}_{E}$	Sink	Time${}_{\mathrm{NE}}$	Time${}_{E}$	Sink	Time${}_{\mathrm{NE}}$	Time${}_{E}$	Sink	Time${}_{\mathrm{NE}}$	Time${}_{E}$	Sink	Time${}_{\mathrm{NE}}$	Time${}_{E}$
1	8.67	14.16	11	8.7	13.94	21	8.61	13.94	31	8.63	13.95	41	8.62	13.95
2	8.67	13.94	12	8.68	13.94	22	8.61	13.94	32	8.63	13.95	42	8.62	13.95
3	8.67	13.94	13	8.66	13.94	23	8.61	13.94	33	8.63	13.95	43	8.62	13.95
4	8.7	13.94	14	8.61	13.94	24	8.61	13.94	34	8.63	13.95	44	8.62	13.95
5	8.7	13.94	15	8.61	13.94	25	8.61	13.94	35	8.63	13.95	45	8.62	13.95
6	8.7	13.94	16	8.61	13.94	26	8.61	13.94	36	8.63	13.95	46		
7	8.7	13.94	17	8.61	13.94	27	8.61	13.94	37	8.62	13.95	47		
8	8.67	13.94	18	8.61	13.94	28	8.61	13.94	38	8.62	13.95	48		
9	8.7	13.94	19	8.61	13.94	29	8.61	13.94	39	8.62	13.95	49		
10	8.7	13.94	20	8.61	13.94	30	8.63	13.96	40	8.62	13.95	50		

**Table A3:** Scenario 3: transmission times (in s) for each sink all over the transmission line. (Distance between poles is equal to 50 m, κ=12, η=2, NE means no encryption, E means encryption.)

Sink	Time${}_{\mathrm{NE}}$	Time${}_{E}$	Sink	Time${}_{\mathrm{NE}}$	Time${}_{E}$	Sink	Time${}_{\mathrm{NE}}$	Time${}_{E}$	Sink	Time${}_{\mathrm{NE}}$	Time${}_{E}$	Sink	Time${}_{\mathrm{NE}}$	Time${}_{E}$
1	8.67	14.16	11	8.97	15.71	21	8.61	13.94	31	8.87	15.46	41	8.62	13.95
2	8.89	15.93	12	8.68	13.94	22	8.87	15.71	32	8.61	13.95	42	8.8	14.88
3	8.95	15.71	13	8.66	13.94	23	8.87	15.71	33	8.61	13.95	43	8.84	15.16
4	8.7	13.94	14	8.92	15.71	24	8.61	13.94	34	8.87	15.46	44	8.62	13.95
5	8.7	13.94	15	8.87	15.71	25	8.61	13.95	35	8.87	15.32	45	8.62	13.95
6	8.95	15.71	16	8.61	13.94	26	8.87	15.46	36	8.61	13.95	46		
7	8.92	15.71	17	8.61	13.94	27	8.87	15.46	37	8.61	13.95	47		
8	8.67	13.94	18	8.87	15.71	28	8.61	13.95	38	8.87	15.18	48		
9	8.7	13.94	19	8.87	15.71	29	8.61	13.95	39	8.87	15.18	49		
10	8.97	15.71	20	8.61	13.94	30	8.87	15.46	40	8.61	13.95	50		

**Table A4:** Scenario 4: transmission times (in s) for each sink all over the transmission line. (Distance between poles is equal to 50 m, κ=8, η=3, NE means no encryption, E means encryption.)

Sink	Time${}_{\mathrm{NE}}$	Time${}_{E}$	Sink	Time${}_{\mathrm{NE}}$	Time${}_{E}$	Sink	Time${}_{\mathrm{NE}}$	Time${}_{E}$	Sink	Time${}_{\mathrm{NE}}$	Time${}_{E}$	Sink	Time${}_{\mathrm{NE}}$	Time${}_{E}$
1	8.67	14.16	11	8.97	15.71	21	9.23	18.06	31	8.61	13.95	41	8.87	15.03
2	8.89	15.93	12	8.68	13.94	22	9.23	18.06	32	8.87	15.32	42	8.61	13.95
3	9.21	18.8	13	8.66	13.94	23	8.87	15.46	33	9.23	17.91	43	8.62	13.95
4	9.27	18.59	14	8.92	15.71	24	8.61	13.95	34	9.23	17.51	44	8.8	14.88
5	8.95	15.71	15	9.27	18.59	25	8.61	13.95	35	8.87	15.18	45	9.12	17.21
6	8.7	13.94	16	9.23	18.59	26	8.87	15.46	36	8.61	13.95	46		
7	8.7	13.94	17	8.87	15.71	27	9.23	18.06	37	8.61	13.95	47		
8	8.92	15.71	18	8.61	13.94	28	9.23	18.06	38	8.87	15.16	48		
9	9.25	18.59	19	8.61	13.95	29	8.87	15.46	39	9.23	17.49	49		
10	9.32	18.59	20	8.87	15.46	30	8.61	13.95	40	9.23	17.36	50		

**Table A5:** Scenario 5: transmission times (in s) for each sink all over the transmission line. (Distance between poles is equal to 50 m, κ=1, η=45, NE means no encryption, E means encryption.)

Sink	Time${}_{\mathrm{NE}}$	Time${}_{E}$	Sink	Time${}_{\mathrm{NE}}$	Time${}_{E}$	Sink	Time${}_{\mathrm{NE}}$	Time${}_{E}$	Sink	Time${}_{\mathrm{NE}}$	Time${}_{E}$	Sink	Time${}_{\mathrm{NE}}$	Time${}_{E}$
1	8.67	14.02	11	14.76	78.49	21	29.9	250.25	31	54.13	531.74	41	87.43	923.1
2	8.89	15.53	12	15.86	90.99	22	31.91	273.45	32	57.04	565.93	42	91.25	968.28
3	9.18	18.13	13	17.06	104.18	23	34.01	297.76	33	60.05	601.22	43	95.16	1014.55
4	9.56	21.82	14	18.34	118.6	24	36.21	323.16	34	63.15	637.61	44	99.17	1061.93
5	10.03	26.62	15	19.72	134.12	25	38.49	349.67	35	66.34	675.1	45	103.27	1110.41
6	10.6	32.51	16	21.19	150.74	26	40.87	377.27	36	69.62	713.69	46		
7	11.25	39.51	17	22.76	168.44	27	43.34	405.98	37	73.0	753.38	47		
8	12.01	47.6	18	24.42	187.24	28	45.9	435.77	38	76.47	794.17	48		
9	12.83	56.8	19	26.16	207.14	29	48.56	466.66	39	80.03	836.05	49		
10	13.75	67.1	20	27.99	228.15	30	51.31	498.65	40	83.68	879.02	50		

### Appendix A.2. Results for the Distance Equal to 200 m

**Table A6:** Scenario 1: transmission times (in s) for each sink all over the transmission line. (Distance between poles is equal to 200 m, κ=2, η=22,η=23, NE means no encryption, E means encryption.)

Sink	Time${}_{\mathrm{NE}}$	Time${}_{E}$	Sink	Time${}_{\mathrm{NE}}$	Time${}_{E}$	Sink	Time${}_{\mathrm{NE}}$	Time${}_{E}$	Sink	Time${}_{\mathrm{NE}}$	Time${}_{E}$	Sink	Time${}_{\mathrm{NE}}$	Time${}_{E}$
1	8.72	9.97	11	14.67	74.56	21	29.7	249.03	31	8.42	39.46	41	21.41	180.97
2	8.91	11.5	12	15.76	87.07	22	31.71	272.53	32	9.31	48.66	42	23.21	201.16
3	9.18	14.1	13	16.94	100.68	23	4.61	5.41	33	10.29	58.97	43	25.1	222.46
4	9.52	17.81	14	18.21	115.38	24	4.76	5.94	34	11.37	70.38	44	27.08	244.85
5	9.98	22.61	15	19.57	131.17	25	5.0	7.31	35	12.54	82.89	45	29.16	268.35
6	10.53	28.52	16	21.03	148.06	26	5.34	9.92	36	13.8	96.5	46		
7	11.18	35.53	17	22.58	166.06	27	5.77	13.63	37	15.14	111.19	47		
8	11.91	43.64	18	24.22	185.15	28	6.29	18.43	38	16.57	126.99	48		
9	12.74	52.85	19	25.95	205.34	29	6.91	24.34	39	18.09	143.88	49		
10	13.66	63.15	20	27.78	226.64	30	7.62	31.35	40	19.7	161.87	50		

**Table A7:** Scenario 2: transmission times (in s) for each sink all over the transmission line. (Distance between poles is equal to 200 m, κ=45, η=1, NE means no encryption, E means encryption.)

Sink	Time${}_{\mathrm{NE}}$	Time${}_{E}$	Sink	Time${}_{\mathrm{NE}}$	Time${}_{E}$	Sink	Time${}_{\mathrm{NE}}$	Time${}_{E}$	Sink	Time${}_{\mathrm{NE}}$	Time${}_{E}$	Sink	Time${}_{\mathrm{NE}}$	Time${}_{E}$
1	8.68	14.17	11	8.72	13.95	21	8.62	13.95	31	8.64	13.96	41	8.63	13.96
2	8.68	13.95	12	8.69	13.95	22	8.62	13.95	32	8.64	13.96	42	8.63	13.96
3	8.68	13.95	13	8.67	13.95	23	8.62	13.95	33	8.64	13.96	43	8.63	13.96
4	8.71	13.95	14	8.62	13.95	24	8.62	13.95	34	8.64	13.96	44	8.63	13.96
5	8.71	13.95	15	8.62	13.95	25	8.62	13.95	35	8.64	13.96	45	8.63	13.96
6	8.71	13.95	16	8.62	13.95	26	8.62	13.95	36	8.64	13.96	46		
7	8.71	13.95	17	8.62	13.95	27	8.62	13.95	37	8.63	13.96	47		
8	8.68	13.95	18	8.62	13.95	28	8.62	13.95	38	8.63	13.96	48		
9	8.72	13.95	19	8.62	13.95	29	8.62	13.95	39	8.63	13.96	49		
10	8.72	13.95	20	8.62	13.95	30	8.64	13.97	40	8.63	13.96	50		

**Table A8:** Scenario 3: transmission times (in s) for each sink all over the transmission line. (Distance between poles is equal to 200 m, κ=12, η=2, NE means no encryption, E means encryption.)

Sink	Time${}_{\mathrm{NE}}$	Time${}_{E}$	Sink	Time${}_{\mathrm{NE}}$	Time${}_{E}$	Sink	Time${}_{\mathrm{NE}}$	Time${}_{E}$	Sink	Time${}_{\mathrm{NE}}$	Time${}_{E}$	Sink	Time${}_{\mathrm{NE}}$	Time${}_{E}$
1	8.68	14.17	11	8.99	15.74	21	8.62	13.95	31	8.9	15.49	41	8.63	13.96
2	8.92	15.95	12	8.69	13.95	22	8.9	15.74	32	8.62	13.96	42	8.82	14.91
3	8.97	15.74	13	8.67	13.95	23	8.9	15.74	33	8.62	13.96	43	8.87	15.18
4	8.71	13.95	14	8.95	15.74	24	8.62	13.95	34	8.9	15.49	44	8.63	13.96
5	8.71	13.95	15	8.9	15.74	25	8.62	13.96	35	8.9	15.34	45	8.63	13.96
6	8.97	15.74	16	8.62	13.95	26	8.9	15.49	36	8.62	13.96	46		
7	8.94	15.74	17	8.62	13.95	27	8.9	15.49	37	8.62	13.96	47		
8	8.68	13.95	18	8.9	15.74	28	8.62	13.96	38	8.9	15.2	48		
9	8.72	13.95	19	8.9	15.74	29	8.62	13.96	39	8.9	15.2	49		
10	8.99	15.74	20	8.62	13.95	30	8.9	15.49	40	8.62	13.96	50		

**Table A9:** Scenario 4: transmission times (in s) for each sink all over the transmission line. (Distance between poles is equal to 200 m, κ=8, η=3, NE means no encryption, E means encryption.)

Sink	Time${}_{\mathrm{NE}}$	Time${}_{E}$	Sink	Time${}_{\mathrm{NE}}$	Time${}_{E}$	Sink	Time${}_{\mathrm{NE}}$	Time${}_{E}$	Sink	Time${}_{\mathrm{NE}}$	Time${}_{E}$	Sink	Time${}_{\mathrm{NE}}$	Time${}_{E}$
1	8.68	14.17	11	8.99	15.74	21	9.26	18.09	31	8.62	13.96	41	8.9	15.05
2	8.92	15.95	12	8.69	13.95	22	9.26	18.09	32	8.9	15.34	42	8.62	13.96
3	9.24	18.84	13	8.67	13.95	23	8.9	15.49	33	9.26	17.95	43	8.63	13.96
4	9.31	18.62	14	8.95	15.74	24	8.62	13.96	34	9.26	17.55	44	8.82	14.91
5	8.97	15.74	15	9.31	18.62	25	8.62	13.96	35	8.9	15.2	45	9.15	17.25
6	8.71	13.95	16	9.26	18.62	26	8.9	15.49	36	8.62	13.96	46		
7	8.71	13.95	17	8.9	15.74	27	9.26	18.09	37	8.62	13.96	47		
8	8.95	15.74	18	8.62	13.95	28	9.26	18.09	38	8.9	15.18	48		
9	9.29	18.62	19	8.62	13.96	29	8.9	15.49	39	9.26	17.52	49		
10	9.36	18.62	20	8.9	15.49	30	8.62	13.96	40	9.26	17.4	50		

**Table A10:** Scenario 5: transmission times (in s) for each sink all over the transmission line. (Distance between poles is equal to 200 m, κ=1, η=45, NE means no encryption, E means encryption.)

Sink	Time${}_{\mathrm{NE}}$	Time${}_{E}$	Sink	Time${}_{\mathrm{NE}}$	Time${}_{E}$	Sink	Time${}_{\mathrm{NE}}$	Time${}_{E}$	Sink	Time${}_{\mathrm{NE}}$	Time${}_{E}$	Sink	Time${}_{\mathrm{NE}}$	Time${}_{E}$
1	8.68	14.03	11	14.89	78.63	21	30.16	250.5	31	54.5	532.11	41	87.92	923.59
2	8.91	15.56	12	16.0	91.13	22	32.18	273.72	32	57.43	566.31	42	91.76	968.78
3	9.21	18.16	13	17.21	104.34	23	34.29	298.04	33	60.45	601.61	43	95.68	1015.07
4	9.6	21.87	14	18.51	118.77	24	36.5	323.45	34	63.56	638.02	44	99.7	1062.46
5	10.09	26.68	15	19.9	134.3	25	38.79	349.97	35	66.76	675.52	45	103.81	1110.95
6	10.67	32.58	16	21.39	150.93	26	41.18	377.59	36	70.06	714.12	46		
7	11.34	39.59	17	22.96	168.64	27	43.67	406.31	37	73.45	753.83	47		
8	12.1	47.7	18	24.63	187.46	28	46.24	436.11	38	76.93	794.63	48		
9	12.94	56.91	19	26.39	207.37	29	48.91	467.01	39	80.5	836.52	49		
10	13.87	67.22	20	28.23	228.39	30	51.67	499.01	40	84.17	879.51	50		

### Appendix A.3. Results for the Distance Equal to 300 m

**Table A11:** Scenario 1: transmission times (in s) for each sink all over the transmission line. (Distance between poles is equal to 300 m, κ=2, η=22,η=23, NE means no encryption, E means encryption.)

Sink	Time${}_{\mathrm{NE}}$	Time${}_{E}$	Sink	Time${}_{\mathrm{NE}}$	Time${}_{E}$	Sink	Time${}_{\mathrm{NE}}$	Time${}_{E}$	Sink	Time${}_{\mathrm{NE}}$	Time${}_{E}$	Sink	Time${}_{\mathrm{NE}}$	Time${}_{E}$
1	8.73	9.98	11	14.71	74.6	21	29.77	249.11	31	8.45	39.49	41	21.47	181.03
2	8.92	11.5	12	15.8	87.12	22	31.79	272.61	32	9.34	48.7	42	23.28	201.23
3	9.19	14.11	13	16.98	100.73	23	4.61	5.41	33	10.33	59.01	43	25.17	222.53
4	9.54	17.82	14	18.26	115.43	24	4.77	5.95	34	11.41	70.42	44	27.16	244.93
5	10.0	22.63	15	19.63	131.22	25	5.01	7.32	35	12.59	82.94	45	29.24	268.43
6	10.55	28.54	16	21.09	148.12	26	5.35	9.93	36	13.85	96.55	46		
7	11.2	35.55	17	22.64	166.12	27	5.79	13.64	37	15.19	111.25	47		
8	11.94	43.67	18	24.28	185.21	28	6.31	18.45	38	16.62	127.04	48		
9	12.77	52.88	19	26.02	205.41	29	6.93	24.36	39	18.15	143.94	49		
10	13.69	63.19	20	27.85	226.71	30	7.64	31.38	40	19.77	161.94	50		

**Table A12:** Scenario 2: transmission times (in s) for each sink all over the transmission line. (Distance between poles is equal to 300 m, κ=45, η=1, NE means no encryption, E means encryption.)

Sink	Time${}_{\mathrm{NE}}$	Time${}_{E}$	Sink	Time${}_{\mathrm{NE}}$	Time${}_{E}$	Sink	Time${}_{\mathrm{NE}}$	Time${}_{E}$	Sink	Time${}_{\mathrm{NE}}$	Time${}_{E}$	Sink	Time${}_{\mathrm{NE}}$	Time${}_{E}$
1	8.68	14.17	11	8.72	13.95	21	8.63	13.95	31	8.64	13.97	41	8.64	13.96
2	8.68	13.95	12	8.7	13.95	22	8.63	13.95	32	8.64	13.97	42	8.64	13.96
3	8.68	13.95	13	8.68	13.95	23	8.63	13.95	33	8.64	13.97	43	8.64	13.96
4	8.71	13.95	14	8.63	13.95	24	8.63	13.95	34	8.64	13.97	44	8.64	13.96
5	8.71	13.95	15	8.63	13.95	25	8.63	13.95	35	8.64	13.97	45	8.64	13.96
6	8.71	13.95	16	8.63	13.95	26	8.63	13.95	36	8.64	13.97	46		
7	8.71	13.95	17	8.63	13.95	27	8.63	13.95	37	8.64	13.97	47		
8	8.69	13.95	18	8.63	13.95	28	8.63	13.95	38	8.64	13.97	48		
9	8.72	13.95	19	8.63	13.95	29	8.63	13.95	39	8.64	13.96	49		
10	8.72	13.95	20	8.63	13.95	30	8.64	13.98	40	8.64	13.96	50		

**Table A13:** Scenario 3: transmission times (in s) for each sink all over the transmission line. (Distance between poles is equal to 300 m, κ=12, η=2, NE means no encryption, E means encryption.)

Sink	Time${}_{\mathrm{NE}}$	Time${}_{E}$	Sink	Time${}_{\mathrm{NE}}$	Time${}_{E}$	Sink	Time${}_{\mathrm{NE}}$	Time${}_{E}$	Sink	Time${}_{\mathrm{NE}}$	Time${}_{E}$	Sink	Time${}_{\mathrm{NE}}$	Time${}_{E}$
1	8.68	14.17	11	9.0	15.74	21	8.63	13.95	31	8.9	15.49	41	8.64	13.96
2	8.92	15.96	12	8.7	13.95	22	8.9	15.74	32	8.63	13.97	42	8.83	14.91
3	8.98	15.74	13	8.68	13.95	23	8.9	15.74	33	8.63	13.97	43	8.88	15.19
4	8.71	13.95	14	8.95	15.74	24	8.63	13.95	34	8.9	15.49	44	8.64	13.96
5	8.71	13.95	15	8.9	15.74	25	8.63	13.97	35	8.9	15.35	45	8.64	13.96
6	8.98	15.74	16	8.63	13.95	26	8.9	15.49	36	8.63	13.97	46		
7	8.95	15.74	17	8.63	13.95	27	8.9	15.49	37	8.63	13.96	47		
8	8.69	13.95	18	8.9	15.74	28	8.63	13.97	38	8.9	15.21	48		
9	8.72	13.95	19	8.9	15.74	29	8.63	13.97	39	8.9	15.21	49		
10	9.0	15.74	20	8.63	13.95	30	8.9	15.49	40	8.63	13.96	50		

**Table A14:** Scenario 4: transmission times (in s) for each sink all over the transmission line. (Distance between poles is equal to 300 m, κ=8, η=3, NE means no encryption, E means encryption.)

Sink	Time${}_{\mathrm{NE}}$	Time${}_{E}$	Sink	Time${}_{\mathrm{NE}}$	Time${}_{E}$	Sink	Time${}_{\mathrm{NE}}$	Time${}_{E}$	Sink	Time${}_{\mathrm{NE}}$	Time${}_{E}$	Sink	Time${}_{\mathrm{NE}}$	Time${}_{E}$
1	8.68	14.17	11	9.0	15.74	21	9.27	18.1	31	8.63	13.97	41	8.9	15.06
2	8.92	15.96	12	8.7	13.95	22	9.27	18.1	32	8.9	15.35	42	8.63	13.96
3	9.25	18.85	13	8.68	13.95	23	8.9	15.49	33	9.27	17.96	43	8.64	13.96
4	9.32	18.63	14	8.95	15.74	24	8.63	13.97	34	9.27	17.56	44	8.83	14.91
5	8.98	15.74	15	9.32	18.63	25	8.63	13.97	35	8.9	15.21	45	9.16	17.26
6	8.71	13.95	16	9.27	18.63	26	8.9	15.49	36	8.63	13.96	46		
7	8.71	13.95	17	8.9	15.74	27	9.27	18.1	37	8.63	13.96	47		
8	8.96	15.74	18	8.63	13.95	28	9.27	18.1	38	8.9	15.19	48		
9	9.3	18.63	19	8.63	13.97	29	8.9	15.49	39	9.27	17.54	49		
10	9.37	18.63	20	8.9	15.49	30	8.63	13.97	40	9.27	17.41	50		

**Table A15:** Scenario 5: transmission times (in s) for each sink all over the transmission line. (Distance between poles is equal to 300 m, κ=1, η=45, NE means no encryption, E means encryption.)

Sink	Time${}_{\mathrm{NE}}$	Time${}_{E}$	Sink	Time${}_{\mathrm{NE}}$	Time${}_{E}$	Sink	Time${}_{\mathrm{NE}}$	Time${}_{E}$	Sink	Time${}_{\mathrm{NE}}$	Time${}_{E}$	Sink	Time${}_{\mathrm{NE}}$	Time${}_{E}$
1	8.68	14.04	11	14.93	78.67	21	30.23	250.58	31	54.61	532.22	41	88.07	923.74
2	8.92	15.56	12	16.05	91.17	22	32.25	273.8	32	57.54	566.42	42	91.9	968.93
3	9.22	18.17	13	17.26	104.38	23	34.37	298.12	33	60.56	601.73	43	95.83	1015.22
4	9.62	21.88	14	18.56	118.82	24	36.58	323.54	34	63.68	638.14	44	99.85	1062.61
5	10.11	26.69	15	19.96	134.35	25	38.88	350.06	35	66.88	675.64	45	103.97	1111.11
6	10.69	32.61	16	21.44	150.99	26	41.27	377.68	36	70.18	714.25	46		
7	11.36	39.62	17	23.02	168.7	27	43.76	406.4	37	73.58	753.96	47		
8	12.13	47.73	18	24.7	187.52	28	46.34	436.2	38	77.06	794.77	48		
9	12.97	56.94	19	26.46	207.44	29	49.01	467.11	39	80.64	836.66	49		
10	13.9	67.25	20	28.3	228.46	30	51.77	499.11	40	84.31	879.65	50		
